# Surface morphology-driven gas sensing performance of CdS-modified TiO_2_ thick film: a study based on fractal concepts

**DOI:** 10.1039/d6ra06387j

**Published:** 2026-10-20

**Authors:** Ankit Kumar Vishwakarma, Ajaya Kumar Sharma, Ram Pratap Yadav, Pradip Kumar Priya, Madhvi Yadav, Lallan Yadava

**Affiliations:** a Department of Physics, Rajendra College, Jai Prakash University Chapra-841301 India kv.ankit92@gmail.com; b Department of Physics, Deen Dayal Upadhyaya Gorakhpur University Gorakhpur-273009 India; c Department of Physics, Allahabad Degree College, University of Allahabad Prayagraj-211003 India; d Department of Physics, Ewing Christian College, University of Allahabad Prayagraj-211003 India aurampratap@gmail.com; e Department of Physics, B. R. M. College, Munger University Munger-841301 India

## Abstract

In recent years, gas sensors have played a crucial role in gas detection technologies, largely due to the increased surface area of their sensing films. The concept of fractals was applied to quantify surface characteristics such as roughness, surface area, and fractal dimension. This approach was employed to enhance sensing performance by informing the design of sensor microstructures. In this study, we investigate how CdS incorporation into TiO_2_ affects the fractal characteristics of the fabricated films and analyse the correlation between these properties and performance in detecting volatile organic compounds (VOCs). Thick films based on TiO_2_ and CdS–TiO_2_ composites were characterised using atomic force microscopy (AFM) and field-emission scanning electron microscopy (FESEM) to analyse their surface morphology. The AFM images were used to estimate parameters such as fractal dimension, Hurst exponent, and surface roughness. The study reveals that these fractal parameters significantly influence the gas sensing properties. The study evaluated the sensing performance of both TiO_2_ and CdS–TiO_2_ thick films for test VOC gases (LPG, ethanol, toluene, and acetone) at concentrations of 1000–5000 ppm and low operating temperatures. Also, the fabricated sensor studies the effect of relative humidity on sensing properties. Among the tested gases, the sensor based on 2 wt% CdS–TiO_2_ exhibited the highest response of 71%, with more selectivity (47.7%) towards acetone over LPG, toluene, and ethanol. The experimental findings are in good agreement with theoretical models, confirming the validity of the approach.

## Abstract 2

CdS-modified TiO_2_ thick films were analysed using AFM fractal parameters and evaluated for acetone sensing at 5000 ppm. The 2 wt% CdS–TiO_2_ sensor achieved a 71% response, highlighting the role of surface morphology in gas-sensing performance.

## Graphical abstract

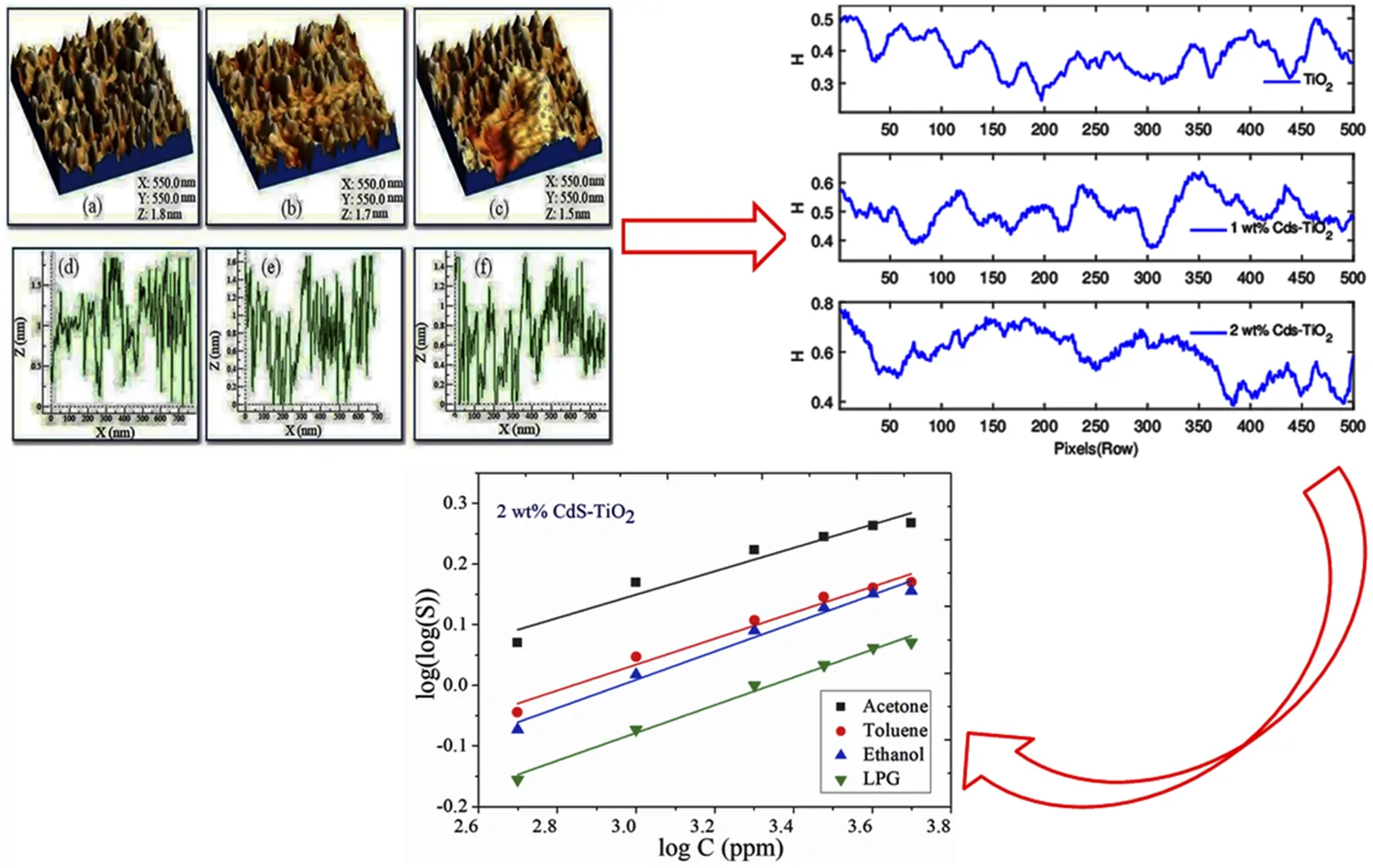

## 1. Introduction

Thin film technology primarily focuses on the fabrication of high-quality structures with tailored surface characteristics, making it applicable across a wide range of scientific and technological fields, including electronics, optics, biomedicine, surface protection, energy, fuel cells, spintronics, magnetism, and sensors.^1,2^ The advancements in thin film deposition techniques have enabled the fabrication of films from a wide range of materials, including metals, non-metals, alloys, ceramics, polymers, and composites. The surface morphology of these films can be effectively analysed using atomic force microscopy (AFM) and scanning electron microscopy (SEM). These powerful characterisation tools provide detailed information about surface texture and structural features at the nanoscale level, which is critical for understanding and optimising the properties of thin films for various applications.^3,4^ Fractal analysis is a powerful technique used to study complex surface structures obtained from AFM and SEM images. Unlike traditional surface analysis methods, fractal analysis can extract additional, in-depth information about surface characteristics.^5,6^ It provides a deeper understanding of how the geometric nature of a surface influences the physical properties of a system.^7,8^ Fractal geometry, a form of non-Euclidean geometry, describes intricate structures characterised by self-similarity across different scales. When applied to gas sensors, such fractal structures offer several key advantages: (i) a large specific surface area, (ii) locally enhanced electric fields, and (iii) improved repeatability of sensor performance. During the deposition process, films with fractal-like structures can form as a result of nanoparticle diffusion and aggregation, which significantly enhance gas sensing performance. Metal oxide semiconductor (MOS) based gas sensors are highly attractive for use in a widespread array of gas sensing applications, and the surface-adsorbed oxygen does play a central role in determining the sensing behaviour.^9^ The development of metal oxide semiconductor (MOS) sensing devices for the detection of volatile organic compounds (VOCs) such as ethanol, liquefied petroleum gas (LPG), acetone, and methanol has become increasingly important in the chemical industry, environmental monitoring, and human health applications.^2^ Acetone, a highly flammable solvent, is widely used as a primary ingredient in nail polish removers and in solvent systems for furniture finishing. Although it evaporates rapidly, acetone is associated with chronic toxicity and can lead to atmospheric oxygen depletion due to its severe oxidation behaviour. To detect acetone and other VOCs, various MOS materials such as ZnO, SnO_2_, WO_3_, and TiO_2_ have been extensively employed. These materials, when fabricated as thin films, offer advantages such as low cost, long lifespan, high sensitivity, and superior selectivity compared to traditional solid-state gas sensors.^10–13^ Among these materials, titanium dioxide (TiO_2_) has been widely studied due to its promising gas-sensing properties, which can be further enhanced by doping with metals such as Pd, Pt, and Ni. Such doped TiO_2_ structures exhibit improved sensitivity to hydrogen and hydrogen-containing molecules.^14,15^ There are numerous studies that have explored TiO_2_-based thick/thin film sensors for detecting alcohols, hydrogen, LPG, and acetone.^16–19^ Bindra *et al.*^20^ performed impedance analysis on TiO_2_-based vapor sensors for methanol and acetone and reported that the sensors demonstrated both resistive and capacitive sensing modes at room temperature. A Stearz *et al.*^21^ reported the dominant role of heterojunctions in gas sensing with composite materials. They found that the contact between the materials plays the dominant role in the sensor response, and it shows the viability of mechanical mixing for composite sample preparation. Park *et al.*^22^ synthesized TiO_2_-functionalized In_2_O_3_ nanowires *via* spin coating and thermal annealing for acetone detection, targeting potential applications in diabetes diagnosis. Yadava *et al.*^23^ reported that a Pd/TiO_2_/Si (Pdtisin) MOS sensor is effective for hydrogen gas detection. In a subsequent study, Yadava *et al.*^24^ fabricated a gridded Pd/TiO_2_/Si sensor for the detection of hydrocarbons such as acetone, ethanol, and trichloroethylene. These investigations highlight the critical role of microstructure in enhancing the gas sensing performance of TiO_2_. Key factors such as grain size refinement, optical bandgap tuning, and control over volume fraction within the films are vital in optimising sensor performance.^25–27^ Vishwakarma *et al.*^28^ reported on XANES analysis and structural properties of CdS-doped TiO_2_. They observed that XANES spectra of the Ti L edges and O K edges of the CdS–TiO_2_ films with 2 wt% CdS doping in the TiO_2_ showed crystal structure changes from the anatase type to the rutile type. Also, they found that the XRD shows that the crystallite size of the TiO_2_ significantly decreases with CdS doping. Refined crystallite/grain size in the 1–2 wt% CdS–TiO_2_ reflects its utility for tailored gas sensing.

The roughness of thin film surfaces significantly influences their physical and chemical properties; therefore, both qualitative and quantitative surface characterization are essential. Surfaces grown under non-equilibrium conditions often exhibit self-affine structures that can be effectively described using fractal concepts and characterised by scaling exponents.^7^ Fractal analysis provides a powerful framework to capture surface features beyond the scope of conventional methods.^29,30^ Among various approaches, Higuchi proposed an efficient algorithm for calculating the fractal dimension of one-dimensional data series.^31^ This method has been widely applied in diverse fields, including electroencephalography (EEG) signal analysis,^32^ the study of external stressors on brain oscillations,^33^ magnetoencephalography (MEG) in Alzheimer's disease patients,^34^ and drowsiness detection methodologies.^35^ Kumar *et al.*^5^ employed the Higuchi algorithm to investigate the effect of heat on the surface morphology of copper (Cu) films, reporting that variations in fractal parameters were correlated with compositional differences between copper oxide phases.

Fractal-structured thin films have shown enhanced properties and superior performance in gas sensing applications. Materials with fractal morphologies, such as SnO_2_ thin films, SnO_2_/CuO composites, TiO_2_, α-Fe_2_O_3_, ZnO, tungsten oxide, and bismuth vanadate, have been successfully applied in sensing technologies.^2^ In these systems, the fractal dimension serves as a critical parameter for quantifying surface roughness.^36,37^ Recent studies further highlight the importance of fractal analysis in thin film characterisation. For example, Das *et al.*^38^ reported a strong correlation between fractal dimension and wettability in aluminium-doped titanium nitride (Ti, Al) N thin films grown by DC and RF magnetron sputtering. Tălu *et al.*^39^ investigated the stereometric and fractal characteristics of sputtered Ag–Cu thin films, establishing a relationship between electrical resistivity and fractal dimension. Similarly, Guisbiers *et al.*^40^ revealed a direct correlation between residual stress and surface complexity in metal thin films. Wang *et al.*^41^ applied multifractal analysis to surface EMG signals, demonstrating a link between muscle fatigue thresholds and fractal dimension.

Atomic force microscopy (AFM) enables direct visualization of temperature- and environment-dependent morphological variations in thin films. Fractal parameters such as interface width, Hurst exponent, and fractal dimension, often computed using Higuchi's algorithm, provide critical insights into these changes. For instance, the fractal characterization of Cu-doped Sr(OH)_2_ thin films revealed that electrochemical performance improved with increasing fractal dimension and decreasing Hurst exponent, with the 3 wt% Cu-doped sample exhibiting anti-persistent behaviour.^8^ Similarly, Yadav *et al.*^6^ reported that Fe doping in Sr(OH)_2_ thin films led to enhanced electrochemical performance, attributed to higher fractal dimensions. These studies demonstrate that fractal analysis offers a non-destructive and effective tool for designing high-performance, cost-efficient, and environmentally friendly functional thin film materials.

In the present study, Higuchi's algorithm has been employed to extract fractal parameters, including the Hurst exponent and fractal dimension, from surface morphology data. The primary objective is to investigate the influence of fractal structures on the gas-sensing properties of CdS–TiO_2_ thick films. By analysing both the surface morphology and sensing performance, we aim to establish a clear correlation between theoretical predictions and experimental observations. This work contributes to the state of the art by demonstrating the practical application of fractal geometry in the design and development of high-performance gas sensors.

## 2. Experimental details and characterisation

### 2.1. Fabrication of TiO_2_ film sensors

Three types of sensors were fabricated on alumina substrates (2.5 cm × 2.5 cm) with a front-side finger electrode pattern and a backside heater pattern. The gas sensors were fabricated using pure TiO_2_, 1 wt% CdS–TiO_2_, and 2 wt% CdS–TiO_2_ compositions. For the preparation of pure TiO_2_ paste, 4.95 g of TiO_2_ powder was manually grinded in a porcelain dish using a top-to-bottom grinding approach with aqueous acetic acid for 30–45 min. Subsequently, 5–10 drops of Laboline binder were added and mixed thoroughly to obtain a homogeneous paste. For the 1 wt% and 2 wt% CdS–TiO_2_ pastes, 0.05 g and 0.10 g of CdS powder were mixed with 4.95 g and 4.90 g of TiO_2_ powder, respectively, followed by the same grinding and paste preparation procedure. The prepared pastes were deposited onto alumina substrates equipped with printed electrodes. The deposited films were subsequently annealed at 523 K for 1 h to improve film adhesion and structural stability. VOCs were used as the target gas to evaluate sensor performance. The required gas concentrations (ppm) were calculated based on the volume of acetone vapour introduced. This setup ensured precise control over the acetone concentration and consistent exposure of the sensors to the target gas. The response of the fabricated sensors to acetone gas was measured under controlled conditions. The sensor was placed inside a test chamber in an air ambient. The chamber allowed for uniform exposure of the sensing film to the target gas while maintaining controlled environmental conditions. Acetone gas at concentrations ranging from 0 to 5000 ppm was introduced into the chamber in an air ambient.

## 3. Characterisation and experimental setup

The surface morphology and grain size of the thick films were analysed using atomic force microscopy (AFM) and Field Emission Scanning Electron Microscopy (FESEM). The AFM was recorded with a Digital Instruments NanoScope IV system. Measurements were conducted in contact mode using a Si_3_N_4_ cantilever (100 µm length, 0.58 N m^−1^ force constant). A He–Ne laser with a power output of 1.5 W and an excitation wavelength of 473 nm was used during imaging. Field Emission Scanning Electron Microscopy was performed using a High-Resolution SEM (HR-SEM, Nova Nano SEM 450). The EDAX spectra were obtained with a Team Pegasus integrated EDS-EBSD system equipped with Octane Plus and Hikari Pro detectors.

The electrical resistance of the sensor was continuously monitored using a Digital Multimeter (Aplab 107N) connected to a Dual DC Power Supply (LD-3202). The sensing thick film was deposited on an interdigitated finger electrode pattern, which was screen-printed on the front side of the substrate using a silver conductor paste (FD6176). A heater electrode pattern was printed on the backside using a ruthenium oxide-based resistor paste (NTC 2413 ESL). A schematic diagram of the experimental setup is shown in Fig. 1. The resistance (*R*_a_) was recorded in air before acetone exposure. The resistance in the presence of acetone (*R*_g_) was recorded during gas exposure. All measurements were conducted at room temperature (∼25 °C). This procedure ensured accurate and reproducible measurement of sensor performance under controlled acetone concentrations. The gas sensing measurements were performed in a closed chamber with a volume of ∼2 L at room temperature (∼300 K). Acetone gas concentration (in ppm) was estimated using the relation based on the injected liquid volume and chamber volume. The sensor was exposed to acetone vapour for a fixed duration, followed by a recovery period in ambient air to restore baseline resistance. The baseline resistance was stable with negligible drift.

**Fig. 1:**
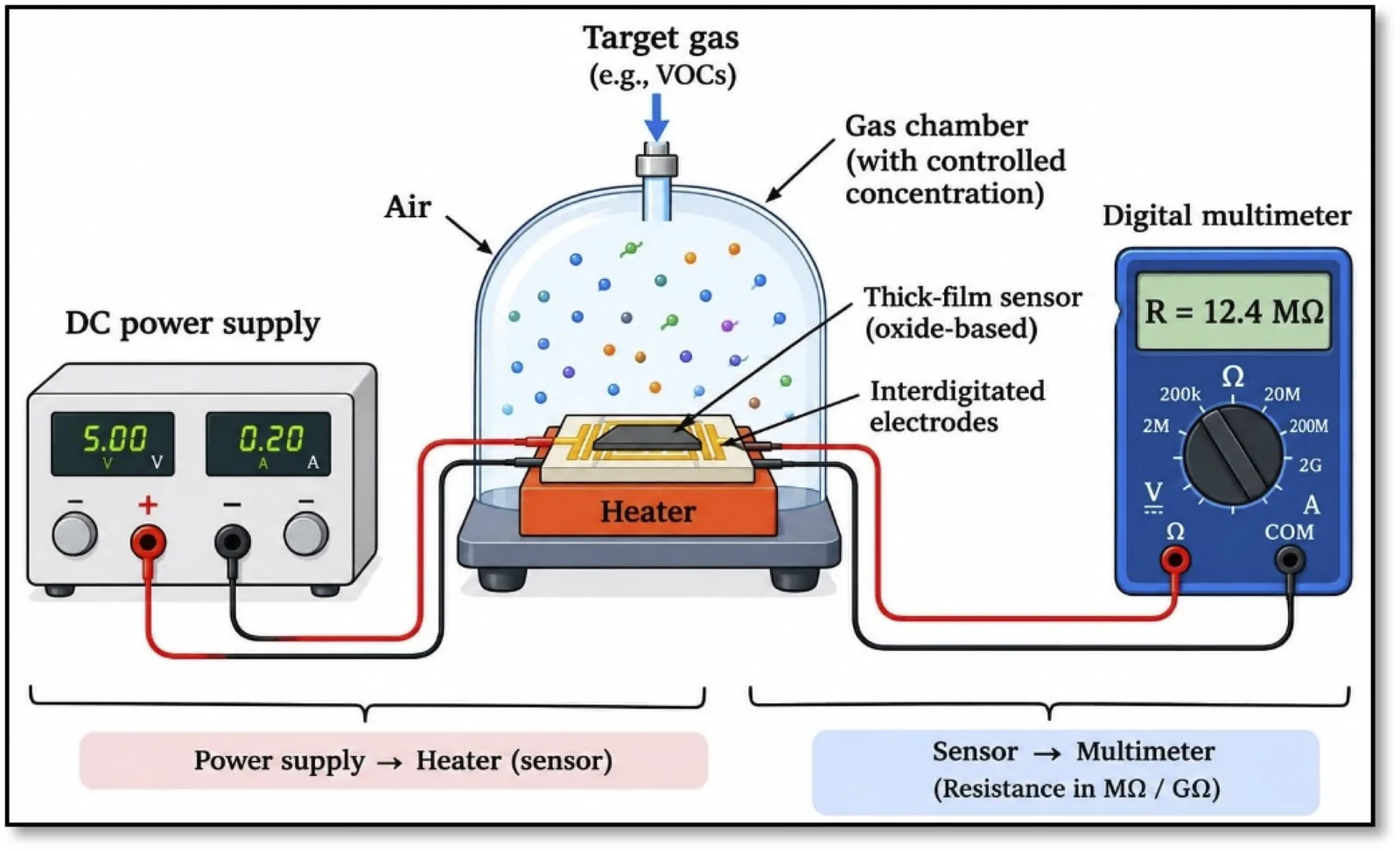
Schematic diagram of the experimental setup used for gas sensing measurements.

## 4. Methods

### 4.1. Roughness measurement

Height parameters characterise the distribution of surface morphological irregularities. One significant parameter is the root mean square deviation from the base plane, commonly referred to as the interface width, mathematically defined as:1
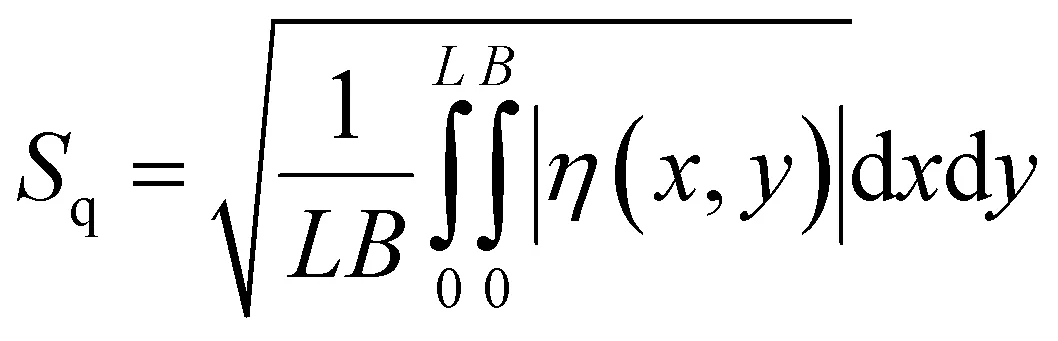
where *η*(*x*,*y*) = *z*(*x*,*y*) − *z*_0_(*x*,*y*), and *η*(*x*,*y*) represents the height deviation of the actual surface *z*(*x*,*y*) from the base plane *z*_0_(*x*,*y*), and *L* and *B* are the length and width of the surface area under consideration. In our case, the base plane is the mean surface from which deviations (roughness) are measured. It is given by 
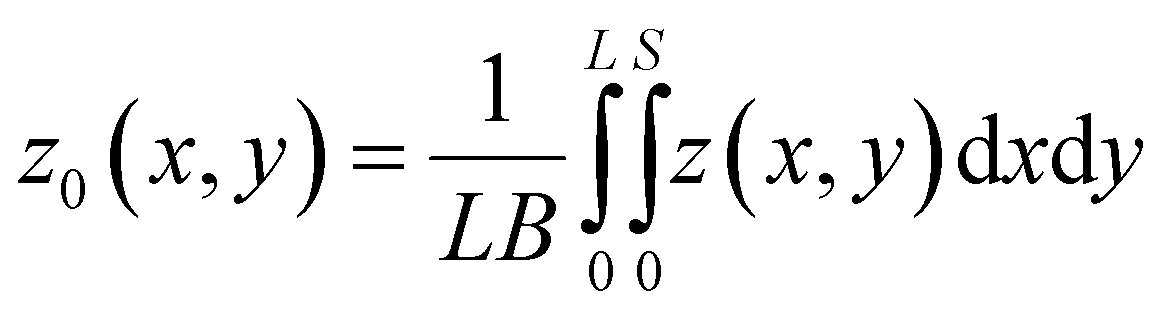
. The value of *S*_q_ is particularly sensitive to extreme peaks and valleys on the surface, providing a robust measure of surface roughness.

### 4.2. Skewness and Kurtosis

The third statistical moment, known as skewness (*S*_sk_), characterises the symmetry of the height distribution of a surface. A negative skewness indicates a surface dominated by valleys, whereas a positive skewness corresponds to a surface with prominent peaks. Skewness is mathematically defined as:2
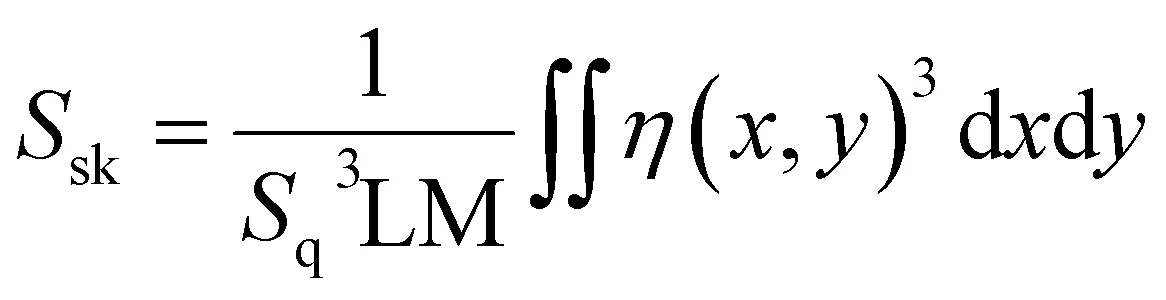


Eqn (2) measures the asymmetry of the height deviations and equals zero for a surface with a normally distributed height profile. A negative skewness indicates that the peak of the height distribution is shifted below the base plane, corresponding to a surface characterised by sharp peaks and broad valleys. Conversely, a positive skewness means the peak of the height distribution is shifted above the base plane, indicating a surface dominated by pronounced peaks.3
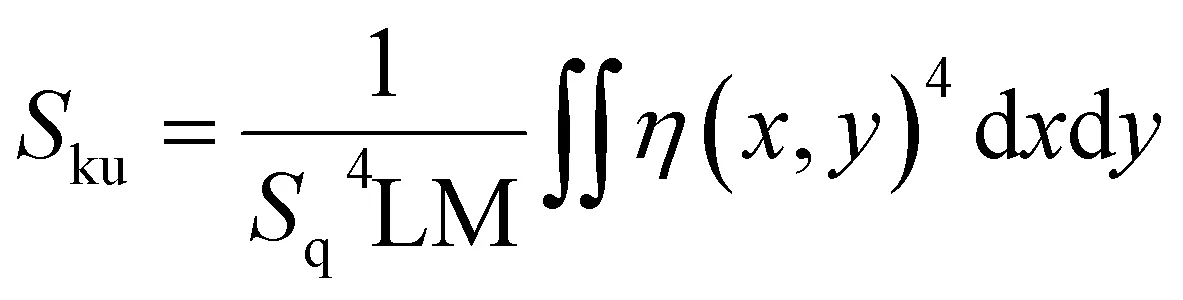


Kurtosis (eqn (3)) is the fourth statistical moment that characterises the flatness or sharpness of the height distribution of a surface. For spiky surfaces, the kurtosis value *S*_ku_ is greater than 3; for bumpy or rounded surfaces, *S*_ku_ is less than 3, and for perfectly random (normal) surfaces, *S*_ku_ equals 3. Essentially, kurtosis measures the sharpness of surface peaks. Higher *S*_ku_ values indicate the presence of extreme peaks and valleys, while lower values suggest a smoother surface morphology. Together, skewness and kurtosis provide valuable information for identifying surface defects such as anomalous peaks or valleys.

### 4.3. Fractal concept and Higuchi algorithm

The formation of fractal structures on the surface of thin films arises from nanoparticle aggregation and diffusion, processes that are strongly influenced by the deposition techniques used. Notably, certain fractal patterns only emerge under specific deposition conditions. Since the nanostructure of these films significantly impacts device performance,^2^ controlling the surface morphology through precise characterization is essential to achieving the desired structural and functional properties. Fractal analysis offers a powerful alternative to conventional surface characterization methods, enabling the extraction of unique surface information. This approach provides deeper insight into the physical properties of thin films, particularly in the context of gas sensing applications. To quantify surface complexity, discretized height data obtained from AFM images can be analysed to calculate the fractal dimension (*D*_f_)) of the film's horizontal cross-sections. The Higuchi algorithm is commonly employed for this purpose, as it offers a simple and efficient means to estimate the fractal dimension directly. The height of each pixel in a horizontal cross-section of the film's surface is represented by a discrete sequence: *X*(1), *X*(2), *X*(3),…, *X*(*N*), where *X*(*i*) denotes the height at the ith pixel, and *N* is the total number of pixels in the section. The *k* sub-sequences have been constructed in the algorithm as

4 *X*_*k*_^*m*^: *X*(*m*), *X*(*m* + *k*), *X*(*m* + 2*k*)………………*X*(*m* + *pk*)

where *m* = 1, 2, 3, …………….*k*, and 
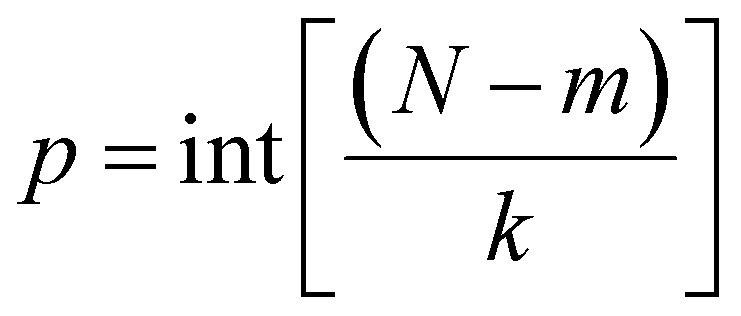
.

The length *L*_*m*_(*k*)of a segment *X*_*k*_^*m*^ of the surface profile is defined as:5
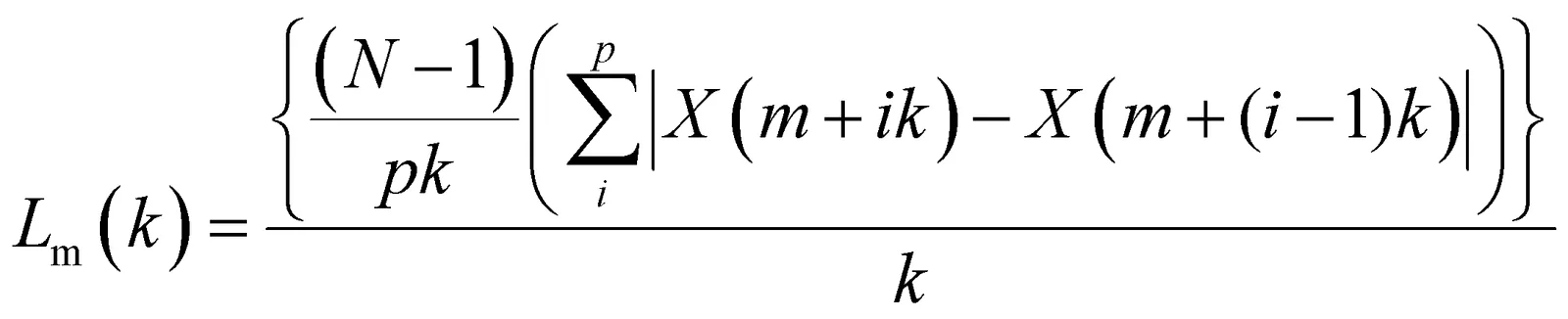
where 
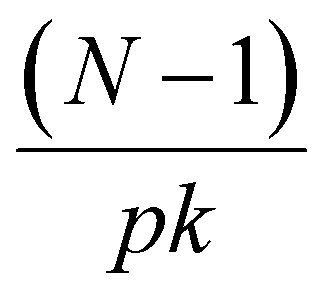
 is a normalisation constant ensuring consistency across different scales.

The average length over all possible segment offsets *m* at scale *k* is given by6
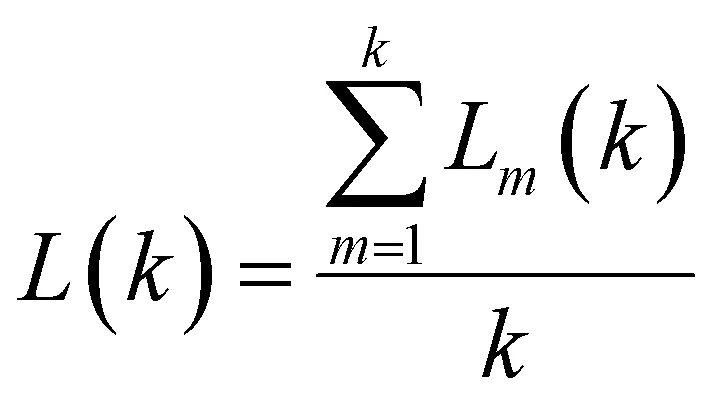


*L*(*k*) scales with *k* according to the power law, which is given by the relation.

7 *L*(*k*) ∼ *k*_f_^−*D*^

Here, *D*_f_ is the fractal dimension. In the context of CdS-doped TiO_2_ thick films, estimating the Hurst exponent from the fractal dimension helps to quantitatively describe how doping affects the surface roughness or texture, which in turn can influence optical, electronic, or catalytic properties. The Hurst exponent *H* is a measure of the long-term memory or roughness of a surface and is related to the fractal dimension *D*_f_ of a surface *via* the following relation:^42,43^

8 *H* = 2 − *D*_f_

The physical significance of *H* is important, as it stands for the measurement of the complexity of the film's surfaces.

### 4.4. Electrical conductivity based on fractal geometry

The pore space is interconnected and saturated with electrical conductivity and a tortuosity factor. This can be expressed as:^44^9
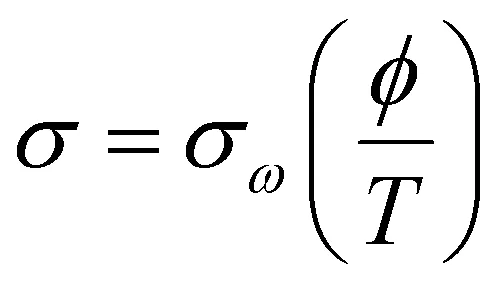
where tortuosity is defined by *T* = *L*_*T*_/*L*_0_, and the value is always ≥1 when surface conductivity is negligible. Here, 
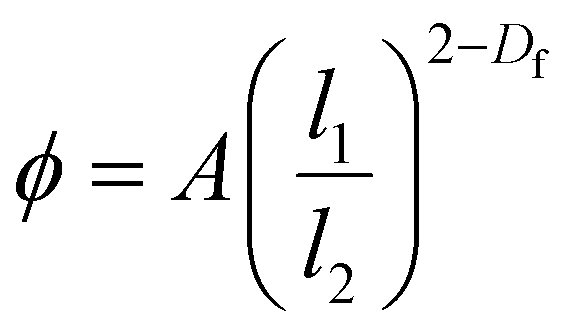
 and *σ* denoted the conductivity, *A* = constant, *l*_1_ = *r*_min_, *l*_2_ = *r*_max_, *r* = proper radius.

Using eqn (8) and (9), we have10
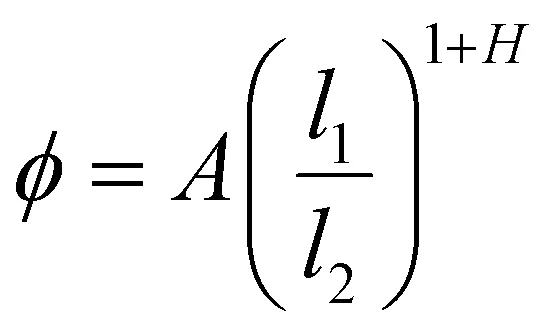
or11
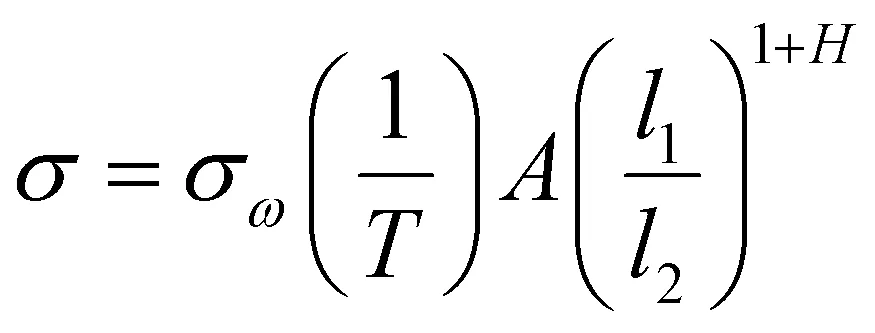


The fractal parameters obtained from AFM analysis provide valuable information about surface morphology and roughness, which influence gas adsorption and charge transport. According to the proposed conductivity model (eqn (9)–(11)), conductivity is related to the Hurst exponent, with an increase in the Hurst exponent corresponding to an increase in conductivity and a decrease in resistance. This relationship suggests that variations in surface fractal characteristics can influence the electrical transport properties and gas sensing response of the material. The theoretical model therefore provides a basis for interpreting the experimentally observed changes in conductivity and resistance in relation to surface morphology.

### 4.5. Response *versus* concentration

It is well known that TiO_2_ sensors operate on the principle of a change in resistance due to the chemisorption of gas molecules on TiO_2_/SnO_2_ surfaces. There is a model predicted by Morrison *et al.*^44^ is used to understand the sensing mechanism. Recently, Mishra *et al.*^45^ proposed models that explain the gas sensing behaviour. Using the Schottky barrier conductance mechanism through the grain boundary and the Freundlich adsorption isotherm, if Δ*R*(=*R*_a_ − *R*_g_) is a change in resistance, then a relation is given by

12 Δ*R* = *A*′exp[*B*′*C*^*b*′^]

where 
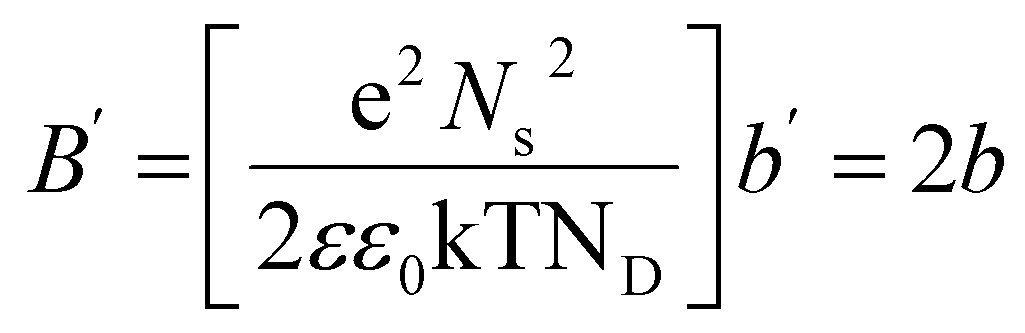
. *R*_a_ is the resistance of the sensor at *C* = 0, and the value of *A*′ = *R*_a_. Putting the value of *A*′ eqn (12) changes as, Δ*R* = *R*_a_ exp[*B*′*C*^*b*′^]13
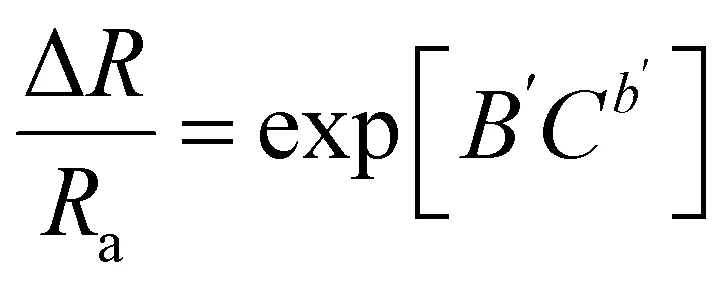


Taking the log on both sides, eqn (13) may be written as: 
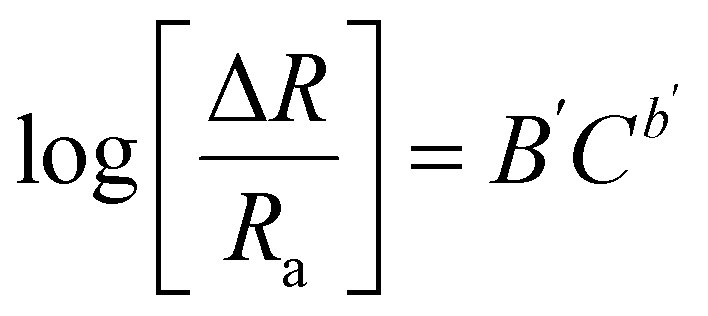
.

Again, taking the log of both sides, we get, log[log(Δ*R*/*R*_a_)] = log(*B*′*C*^*b*′^)

14 log[log(Δ*R*/*R*_a_)] = log(*B*′) + *b*′log(*C*)

Comparing eqn (14) with *Y* = *m*·*X* + *n*, here slop *m* = *b*′ (constant) *n*. The plot of sensing response *vs.* concentration gives a straight line (Section 4.3, Fig. 11).

## 5. Results and discussion

### 5.1. Atomic force microscopy (AFM)

We employed atomic force microscopy to characterise the surface morphology of CdS–TiO_2_ thick films. The AFM images (Fig. 2) show irregular film surfaces with varying grain sizes and non-uniform separations. A smaller grain size is observed with lower CdS content. The surface morphology of both thin and thick films demonstrates a fractal nature,^2^ with self-affine structures being the most prominent. A self-affine surface appears statistically similar under anisotropic scaling (affine transformations). To quantify the fractal characteristics, we applied the Higuchi algorithm, a direct method originally developed to estimate fractal dimensions of time series data, now increasingly used in surface analysis across various disciplines.^2,5–8^ The fractal dimension derived from this method reflects the complexity of the grain structure, which is closely related to the gas sensing performance of the films. The variation in grain size may contribute to the differing fractal behaviours of the films across various scale ranges. The fractal dimension values were estimated using eqn (7), as illustrated in Fig. 3.

**Fig. 2:**
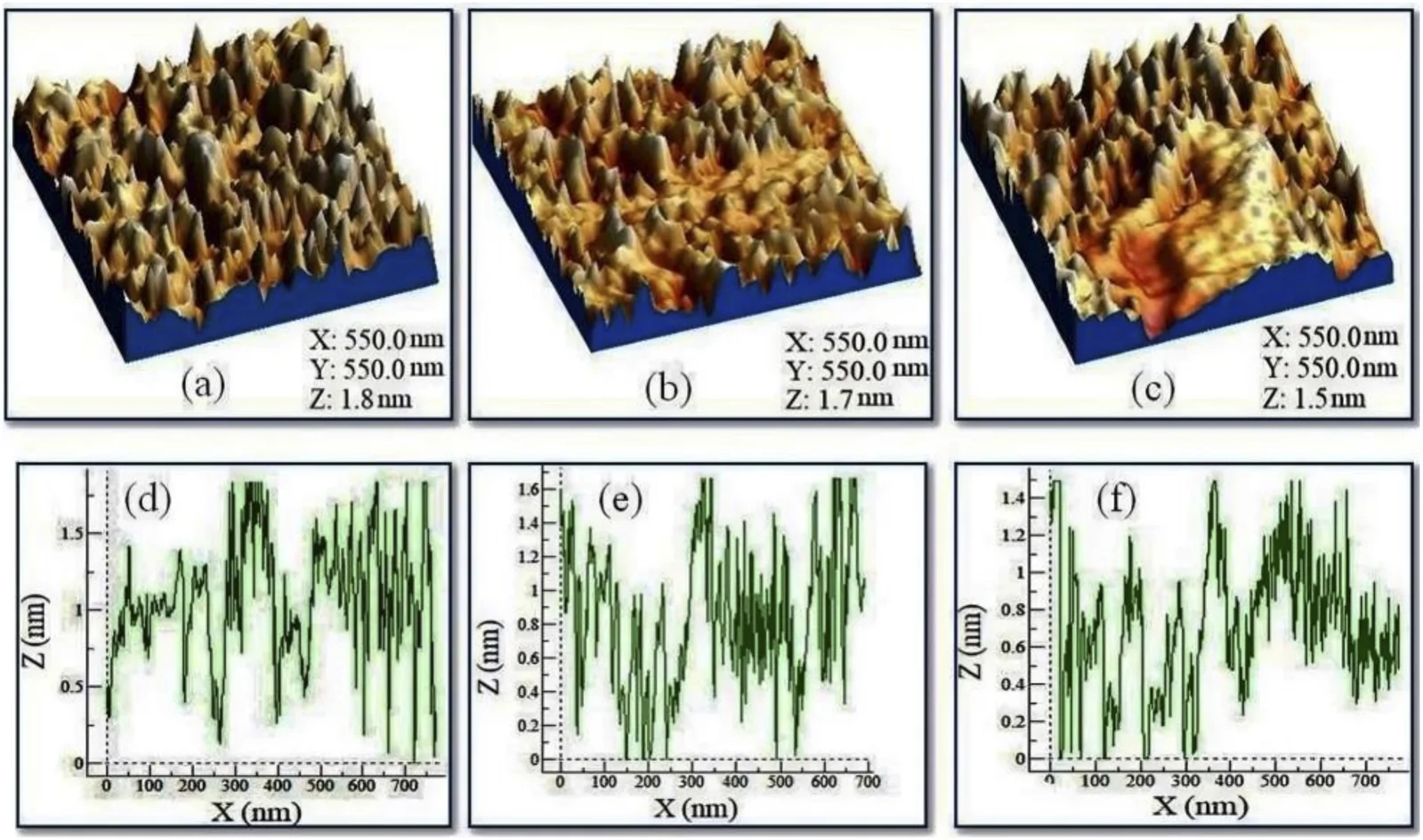
AFM surface Morphology (a) TiO_2,_ (b) 1 wt% CdS–TiO_2,_ (c) 2 wt% CdS–TiO_2,_ and AFM surface profile picture of (d) TiO_2,_ (e) 1 wt% CdS–TiO_2,_ (f) 2 wt% CdS–TiO_2._

**Fig. 3:**
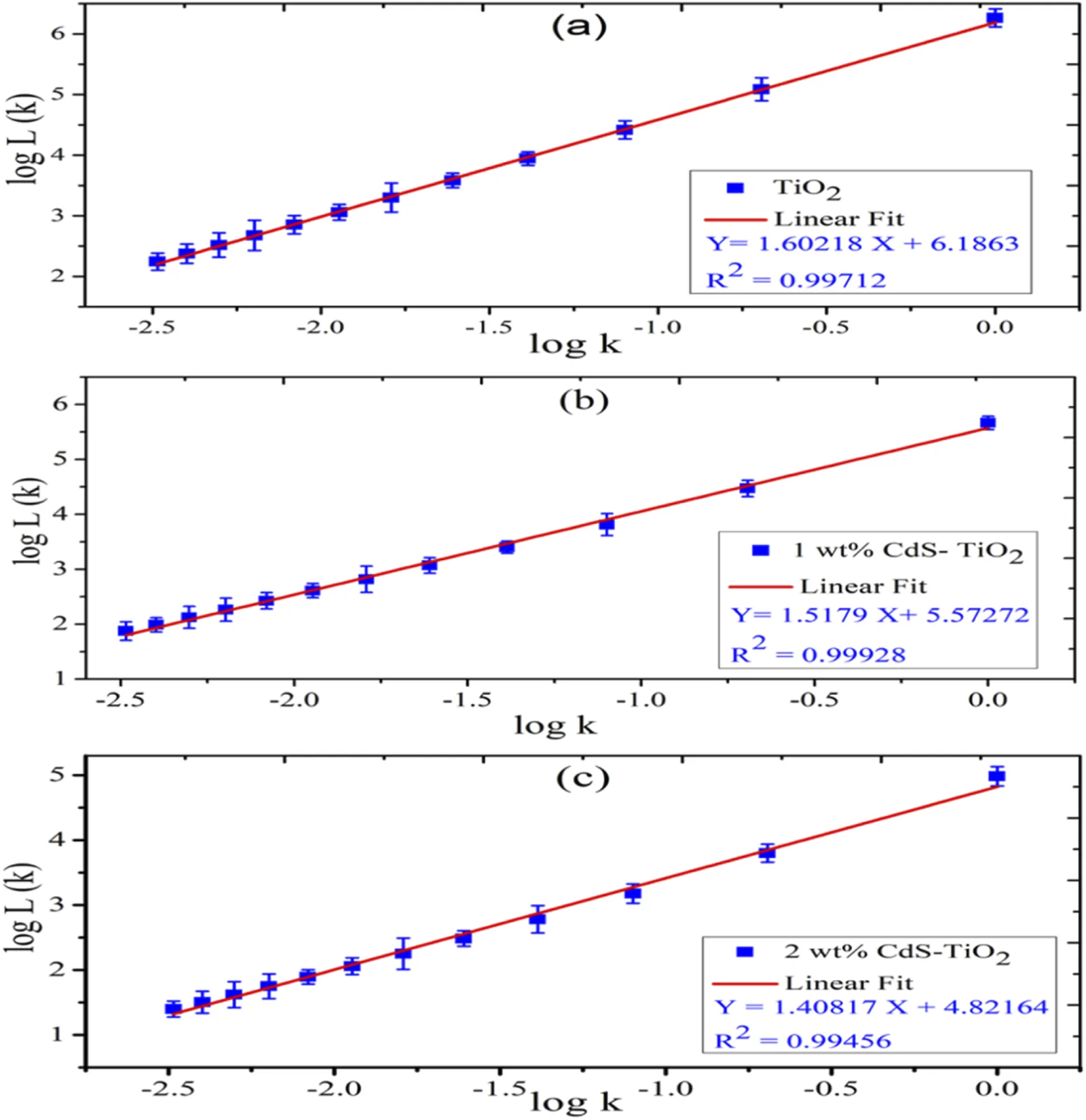
Variation of log *L*(*k*) as a function of log *k* for sensors (a) TiO_2_, (b) 1 wt% CdS–TiO_2_, and (c) 2 wt% CdS–TiO_2_. The red solid lines are the best fit for the respective curves.

Additionally, the Hurst exponent was calculated using eqn (11). The variation of the Hurst exponent with pixel number for all films is presented in Fig. 4. The results show that the roughness increases with 2 wt% CdS content; therefore, the volume surface ratio increases, *i.e.*, more surface is available to release electrons from the TiO_2_ surfaces in comparison to without CdS. Hence, the fabricated sample with 2 wt% CdS–TiO_2_ is suitable for gas sensing applications.

**Fig. 4:**
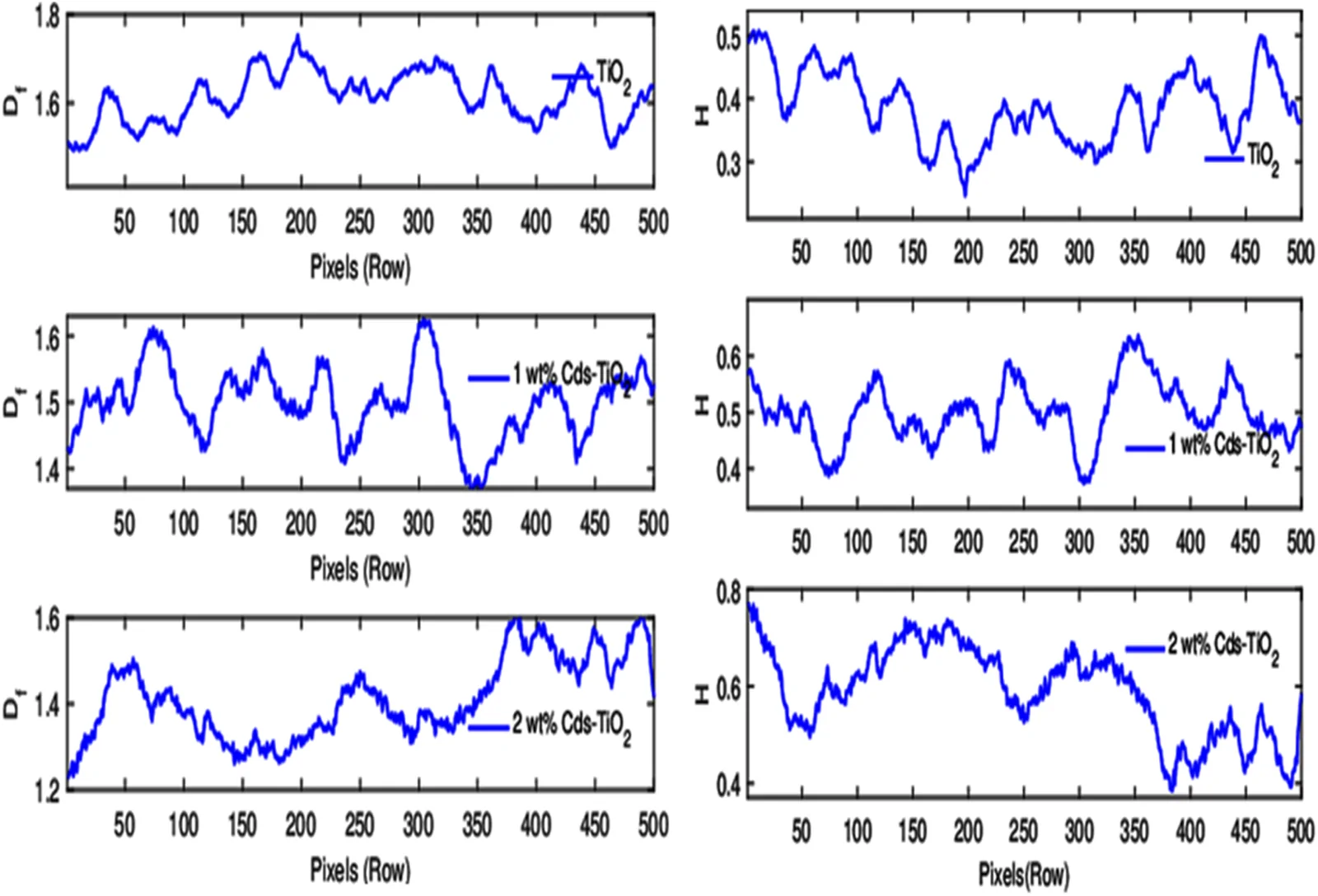
Fractal dimension (*D*_f_) value for row pixels (left side) and Hurst exponent (H) value of row pixels for sensors TiO_2,_ 1 wt% CdS–TiO_2,_ and 2 wt% CdS–TiO_2_ (right side).

Table 1 shows that the Hurst exponent increases with CdS content, and it is greater than 0.5 for 2 wt% CdS–TiO_2_, which indicates that it has the roughest surface. The deposition parameters influencing the fractal structure of the films are closely related to the mechanisms governing fractal pattern formation. The variation of the Hurst exponent (H) as a function of image rows for different CdS doping concentrations is shown in Fig. 4. The average Hurst exponent values for each doping level are summarised in Table 1. The Hurst exponent characterises surface roughness at small scales and generally ranges from 0 to 1.^5,6^ A value of *H* < 0.5 indicates that surface heights at neighbouring pixels are negatively correlated (anti-persistent behaviour), whereas *H* > 0.5 suggests positive correlation (persistent behaviour) between neighbouring heights.^6–8^ For films with 2 wt% CdS content, the Hurst exponent values are greater than 0.5, indicating the presence of persistence in surface growth during deposition. Conversely, films with less than 2 wt% CdS exhibit H values below 0.5, suggesting anti-persistent behaviour. A value of *H* = 0.5 corresponds to an uncorrelated or random surface, which is characteristic of a random walk process,^6^ while *H* > 0.5 implies the presence of a memory effect in the growth dynamics. Thus, it is clear that both types of behaviour exist in the system: memory as well as a non-memory effect during deposition*.* The system has a memory effect and performs better in sensing compared to others. Moreover, Fractal dimension *D*_f_ gives information about the smoothness or roughness of the surface morphology. The higher the value of the fractal dimension, the more irregular the surface morphology.^46^ And so, 2 wt% CdS–TiO_2_ films will have a smoother surface as compared to the 1 wt% CdS–TiO_2_ or 0 wt% CdS–TiO_2_ films.

**Table 1:** Fractal dimensions, Hurst exponent, roughness skewness, kurtosis, grain size slope, and intercept of TiO_2,_ 1 wt% CdS–TiO_2,_ and 2 wt% CdS–TiO_2_ films

CdS (wt%)	Fractal dimension (*D*_f_)	Hurst exponent (*H*)	Roughness	Skewness	Kurtosis	Grain size (nm)
0	1.6119 ± 0.0563	0.3881 ± 0.0563	7.17 ± 0.12	0.0522 ± 0.003	2.531 ± 0.02	29.17 ± 0.45
1	1.5002 ± 0.0523	0.4998 ± 0.0523	5.70 ± 0.34	0.0167 ± 0.002	2.523 ± 0.03	23.74 ± 0.51
2	1.4095 ± 0.0916	0.5905 ± 0.0916	9.03 ± 0.25	0.0125 ± 0.004	2.465 ± 0.01	18.75 ± 0.85

In Table 1, the Hurst exponent increases with CdS content, so conductivity may also increase (resistance decreases) with CdS content in TiO_2_. Hence, these devices are suitable for sensing applications for reducing gases. The surface morphology of gas sensors typically consists of both macro- and microstructures. The sensing performance of these devices is closely correlated with the nanostructure of the films, which is indirectly influenced by the substrate used during fabrication.^9^ The film's microstructure is primarily determined by the choice of gas-sensing material and the deposition technique employed. Fractal geometries, complex and irregular by nature, can be generated through simple iterative processes. Such structures exhibit unique properties and often possess a significantly larger specific surface area compared to conventional Euclidean geometries. Films with fractal surface structures can enhance the performance of gas sensors by providing more active sites for gas interaction. It has been observed that different types of fractal structures exhibit varying properties, which can lead to distinct effects on the overall film characteristics. To quantify these structures, researchers commonly use the fractal dimension.^37^ For certain types of fractal geometries, films with higher fractal dimensions typically have a larger effective surface area. This increased surface area provides more interaction sites for gas molecules, thereby enhancing the gas sensor response.

Grain size plays a critical role in determining the sensitivity of gas-sensing films.^2^ Kante *et al.*^47^ fabricated SnO_2_ thin films consisting primarily of a single layer of nanoaggregates (5–10 nm) using an electrochemical process. They successfully controlled the dendritic fractal structure and its dimension by varying the current density. However, their reported sensor responses to ethanol and 300 ppm CO at 197 °C, 250 °C, and 300 °C were inconsistent with theoretical predictions and needed to be scaled by a factor of 10 for alignment. The study suggested that while fractal-shaped structures increase the specific surface area in contact with gas molecules, they do not directly influence sensitivity, which was found to depend primarily on grain size. This conclusion appears to contradict the commonly accepted theory. In our view, while the nano aggregate structure might cause grain size to dominate sensitivity, other factors, such as surface area, porosity, and morphology, should not be overlooked.

It can be hypothesised that incorporating fractal geometries in gas sensor design could significantly enhance sensitivity by increasing the surface-to-volume ratio, thereby providing a larger active surface area for gas interaction. Indeed, films with fractal structures have been explored to enhance gas sensing performance and are considered promising for developing high-sensitivity sensors. It is also noted that minor surface inhomogeneity may contribute to deviations in the local fractal dimension, and this point will be further investigated in future work through localised fractal analysis of segmented regions. Although fractal parameters such as fractal dimension, Hurst exponent, skewness, and kurtosis provide quantitative insight into surface morphology, their influence on gas sensing behaviour must be interpreted with care. The fractal dimension and Hurst exponent are related to surface irregularity and spatial correlation of surface features, which can affect adsorption and desorption kinetics and electron transport pathways. Increased surface complexity may enhance the density and distribution of active sites for oxygen adsorption, thereby promoting charge transfer during acetone oxidation and improving the sensing response (Section 4.3). However, the present study does not include direct modelling or spectroscopic validation of charge-transfer processes. The observed correlations are primarily based on AFM-derived morphology and sensing measurements. Therefore, fractal parameters are considered as complementary descriptors rather than sole governing factors, and further studies involving controlled morphology and advanced characterisation are required for a more rigorous mechanistic understanding.^48^

### 5.2. FESEM analysis

The FESEM micrographs of the fabricated thick films, undoped TiO_2_, 1 wt% CdS–TiO_2_, and 2 wt% CdS–TiO_2_, are shown in Fig. 5(a–c). As seen in Fig. 5(a), the undoped TiO_2_ film exhibits a dense, continuous, and relatively homogeneous distribution of distinct, spherical nanoparticles, with distributed grains ranging from 29 to 70 nm. A unique interfacial phenomenon is observed in Fig. 5(b). It shows the microtecturing and microgroove structure. The presence of finer grains (23–75 nm) dispersed over the TiO_2_ surfaces confirms the incorporation of CdS nanoparticles into the TiO_2_ matrix. In comparison, the 2 wt% CdS–TiO_2_ thick film demonstrates a more heterogeneous, irregular and more kinked morphology, with hybrid structures distributed across the surface.^49^Fig. 5(c) exhibits smaller grains ranging from 18 to 40 nm. The formation of such hybrid architectures is beneficial, as they provide a greater number of active sites for water molecule adsorption, ultimately improving sensing efficiency. SEM analysis highlights the critical role of surface morphology, grain size, and porosity in determining the performance of CdS–TiO_2_ nanocomposites. A porous architecture ensures a high surface-to-volume ratio, which promotes enhanced water molecule adsorption and improved sensor sensitivity. Furthermore, the interconnected pores enable rapid diffusion of humidity, resulting in faster response and recovery times. The average grain size was calculated, and a particle distribution histogram was plotted from the SEM micrograph. In the statistical fitting graph shown in Fig. 6(a–c), the average particle size is 59 nm, 52 nm and 28 nm for TiO_2_, 1wt% CdS–TiO_2_ and 2 wt% CdS–TiO_2_, respectively.

**Fig. 5:**
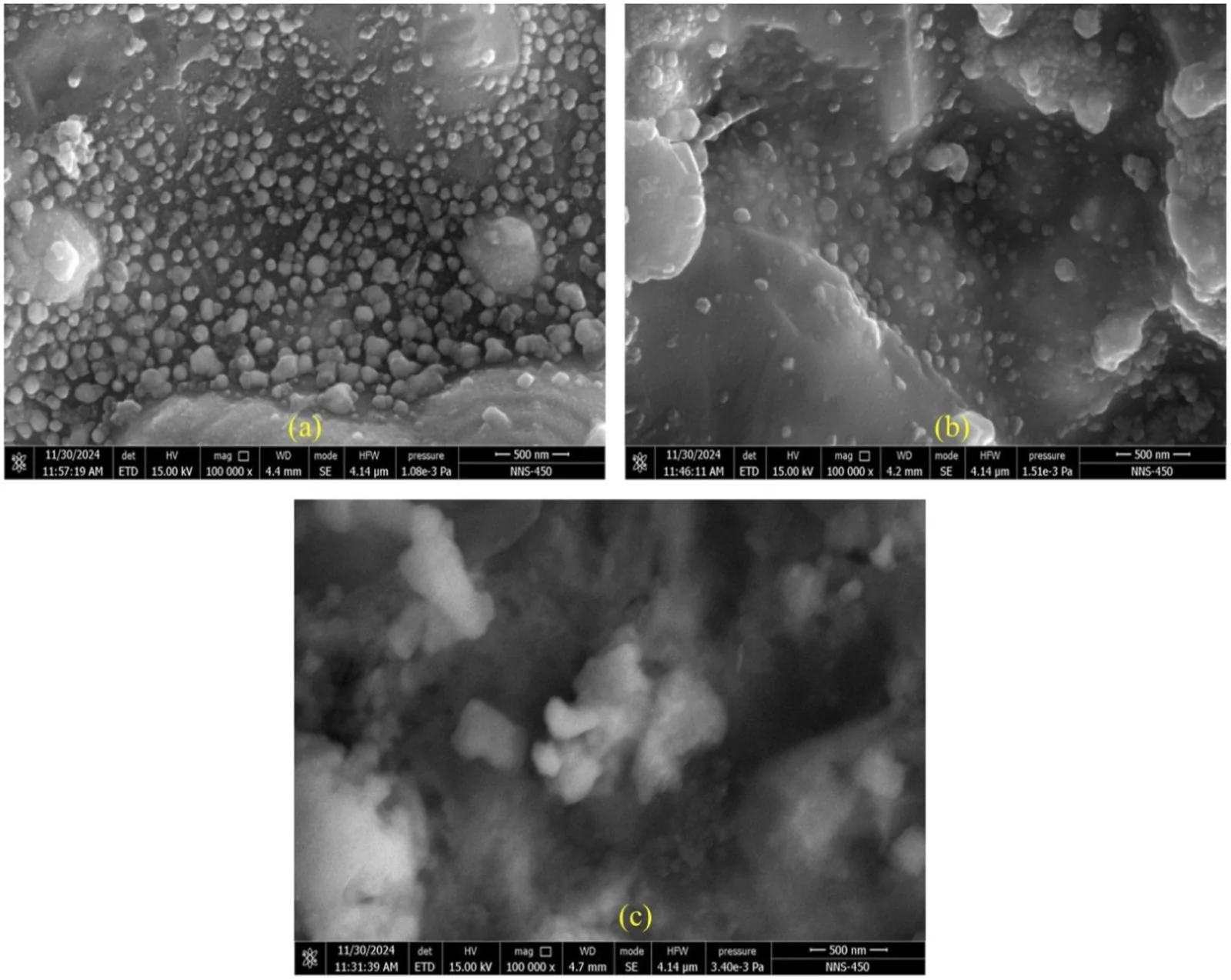
FESEM images of (a) TiO_2_, (b) 1 wt% CdS–TiO_2_, and (c) 2 wt% CdS–TiO_2_ thick films.

**Fig. 6:**
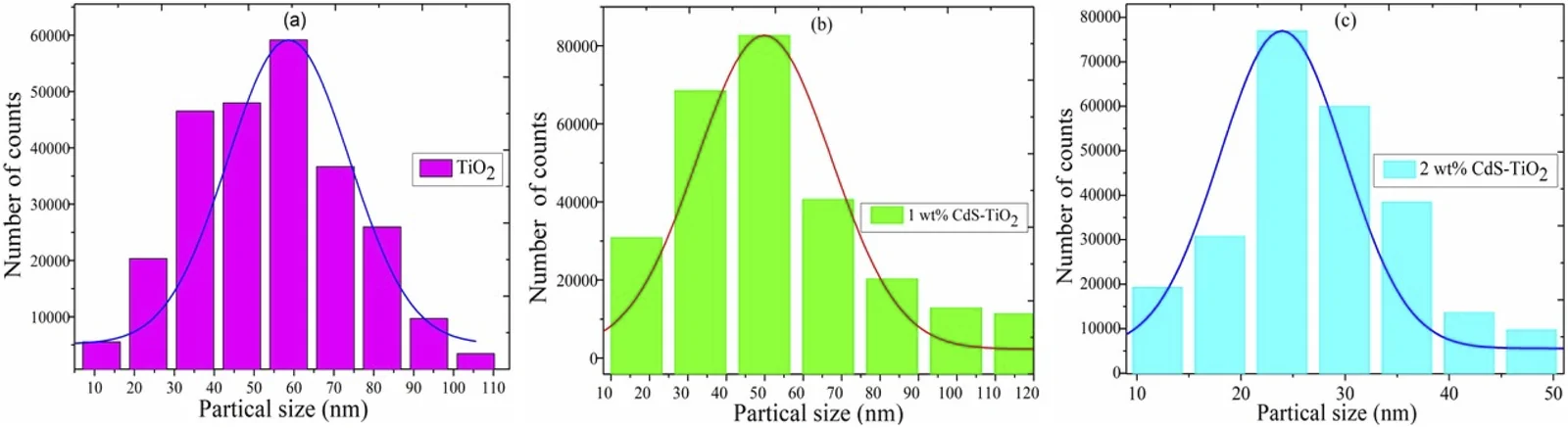
Statistical particle size distribution histogram of the fabricated sensor: (a) TiO_2_, (b) 1 wt% CdS–TiO_2_ and (c) 2 wt% CdS–TiO_2_.

Elemental composition was further investigated using Energy-Dispersive X-ray Analysis (EDAX). The EDAX spectra of TiO_2_ and 1–2 wt% CdS–TiO_2_ are shown in Fig. 7(a–c). The elemental spectra clearly confirm the presence of Ti, O, and CdS in the fabricated films, verifying the successful incorporation of CdS nanoparticles into the TiO_2_ matrix.^50^ In addition, Ag peaks were consistently detected in all three spectra. These peaks arise from the silver finger electrodes utilised in the device configuration and are not associated with the nanocomposite itself.

**Fig. 7:**
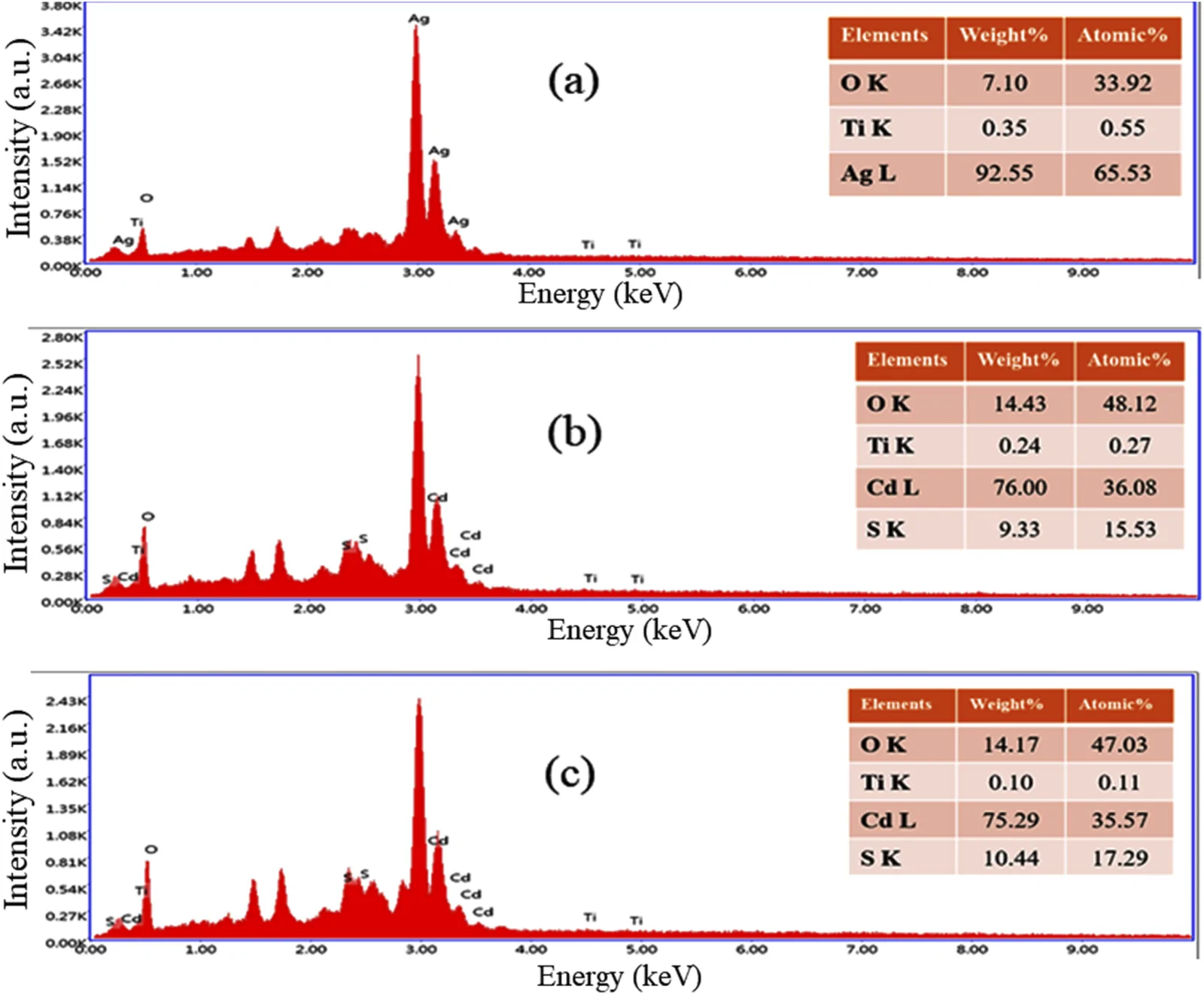
: EDAX spectra of (a) TiO_2_, (b) 1 wt% CdS–TiO_2_, and (c) 2 wt% CdS–TiO_2_ thick films.

The combined SEM and EDAX analyses, therefore, confirm both the morphological modifications introduced by CdS doping and the elemental composition of the fabricated samples. Together, these findings provide strong evidence of successful CdS incorporation into the TiO_2_ thick films and demonstrate the structural features that contribute to improved VOC sensing characteristics.

### 5.3. Gas sensing properties

The gas-sensing performance of the fabricated thick film sensors, TiO_2_, 1 wt% CdS–TiO_2_, and 2 wt% CdS–TiO_2_, was evaluated toward ethanol, LPG, toluene, and acetone. The response (%) of the sensor was calculated using the formula (eqn (15)), where *R*_a_ is the resistance in air, and *R*_g_ is the resistance in the presence of the target gas.^51–53^15
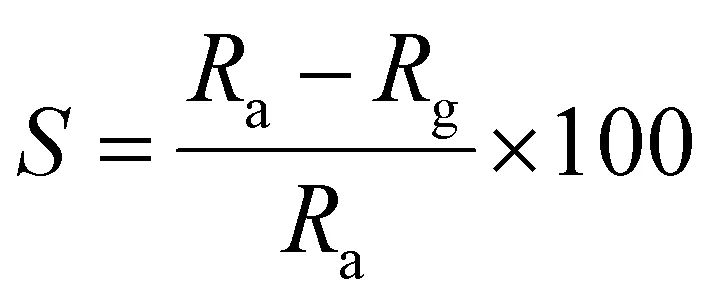


The sensing response (%) measurements were performed at room temperature (300 K) in ambient air with test gas concentrations ranging from 0 to 5000 ppm. The response (%) of the sensor is shown in Fig. 8(a–d); CdS incorporation markedly improved the response for the test gases ethanol, LPG, toluene, and acetone. The maximum responses (Fig. 8d) were ∼71% for 2 wt% CdS–TiO_2_, 43% for 1 wt% CdS–TiO_2_, and 22% for undoped TiO_2_ for acetone. After 5000 ppm, no change in the electrical resistance of the sensor is observed, and the response is almost saturated. These values exceed previously reported responses (2.2–26.0%) for acetone concentrations of 20–500 ppm using nanoporous TiO_2_ at 370 °C and TiO_2_ nanostructures at 100–400 °C.^54^ The response values for other test gases, ethanol, LPG, and toluene, is presented in Table 2.

**Fig. 8:**
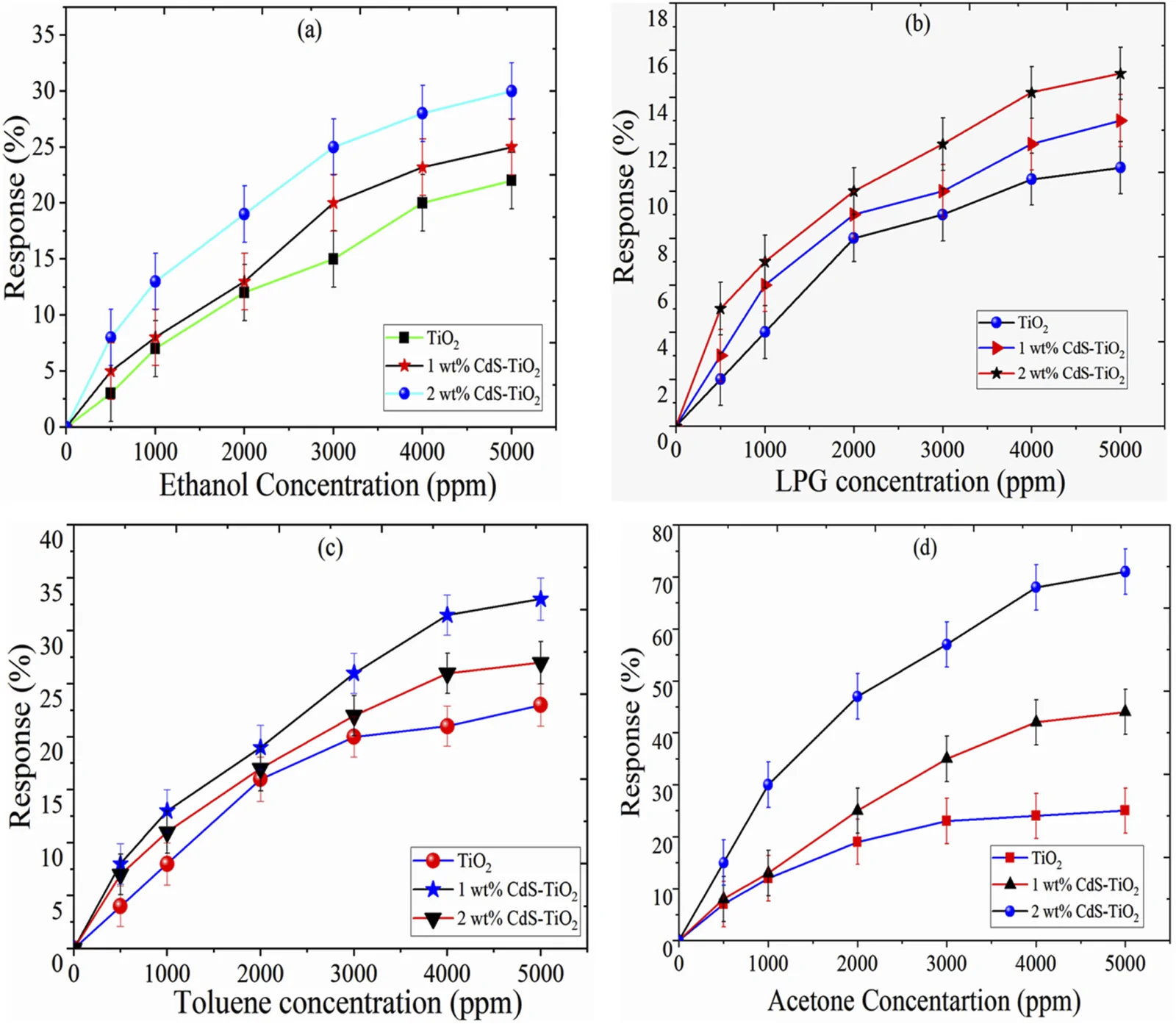
Response (%) of sensors TiO_2,_ 1 wt% CdS–TiO_2,_ and 2 wt% CdS–TiO_2_ for (a) ethanol, (b) LPG, (c) toluene, and (d) acetone with error bars at room temperature (300 K).

**Table 2:** Response (%) of the sensor, selectivity, and transient time of the sensor for ethanol, LPG, toluene, and acetone

Test gases	Response (%)			Transient time (2 wt% CdS–TiO_2_)		Selectivity (%) (2 wt% CdS–TiO_2_)
	TiO_2_	1 wt% CdS–TiO_2_	2 wt% CdS–TiO_2_	Response time (sec)	Recovery time (sec)	
Ethanol	22	25	30	68	177	20.1
LPG	10	13	15	88	212	10.1
Toluene	23	27	33	71	181	22.1
**Acetone**	**22**	**43**	**71**	**50**	**120**	**47.7**

Selectivity is one of the most important parameters of the sensing properties of the developed sensor. The selectivity of the sensor was calculated using the formula^55^16
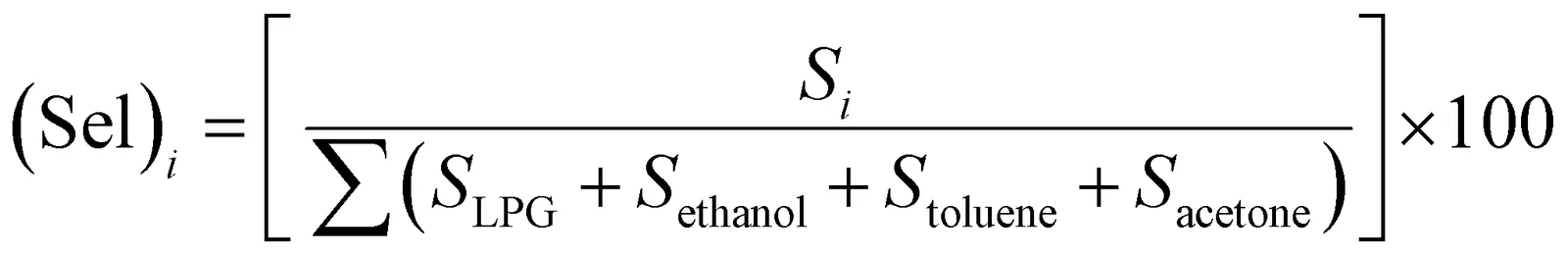
where *S*_i_ is the response of the gas for which the relative selectivity is to be estimated. It is found to be ∼47.7%, ∼22.1%, ∼20.1%, and ∼10.1% for acetone, toluene, ethanol, and LPG, respectively, which is displayed in the pie diagram (Fig. 9). It is evident from Fig. 9 that acetone is more selective than LPG, ethanol, and toluene for 2 wt% CdS–TiO_2_. To provide the actual dynamic sensing behaviour of the fabricated sensor, the experimentally measured sensor resistance (*R*) as a function of time is shown in Fig. 10(a–d), which represents the transient time (response and recovery time). The resistance transient curves clearly demonstrate the reversible resistance changes upon repeated exposure to the target gas and recovery in air, confirming the stability and repeatability of the sensor response. The response time was defined as the duration required for the signal to reach 90% of its final value after gas exposure, whereas the recovery time corresponded to the time needed to return to 10% above the baseline after gas removal.^56^ For 2 wt% CdS–TiO_2_, the response time decreased from 80 s to 50 s, and the recovery time from 195 s to 120 s at 5000 ppm acetone, demonstrating significantly faster response compared to the other test gases. The transient time for LPG, acetone, ethanol, and toluene is shown in Table 2.

**Fig. 9:**
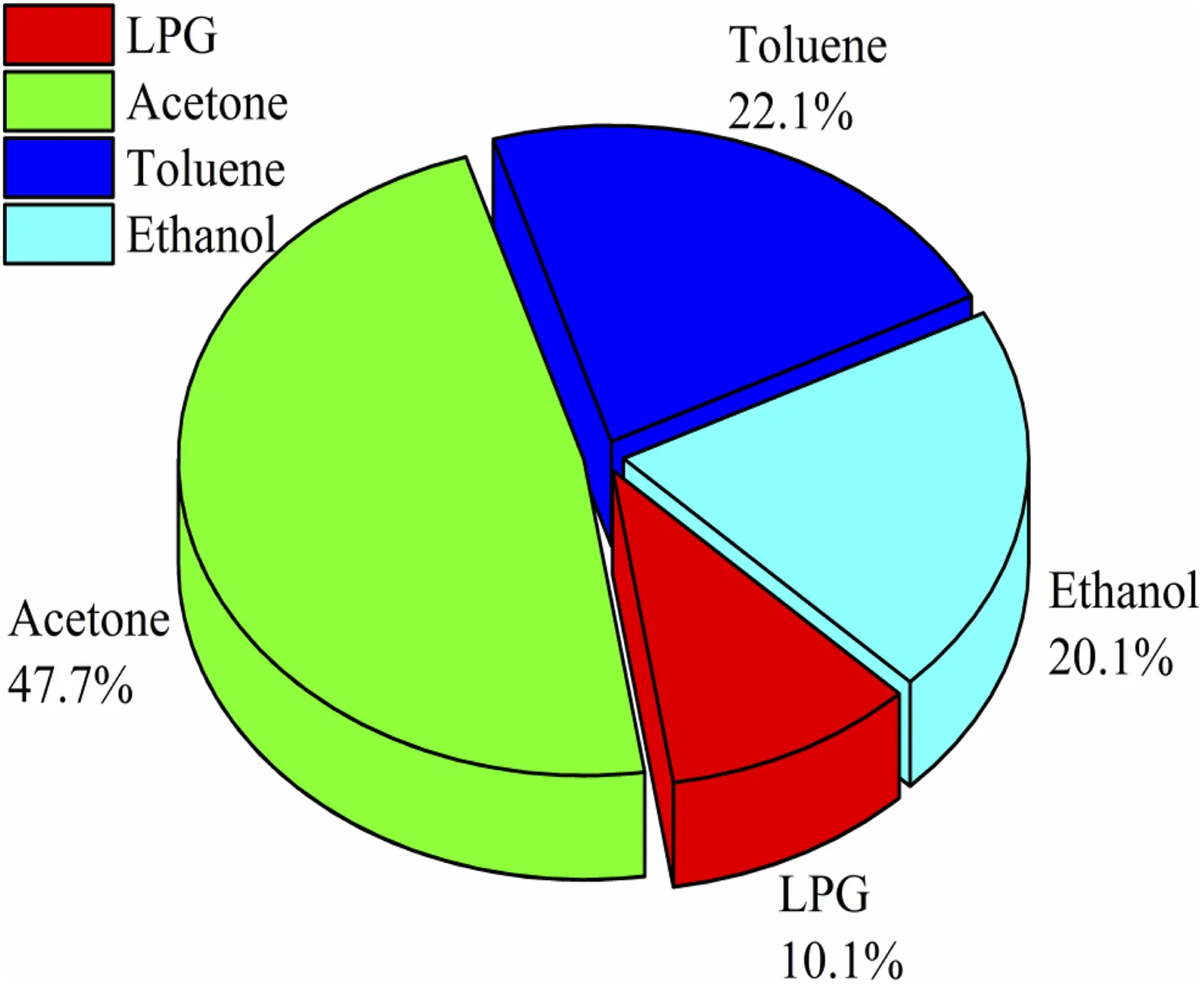
: Selectivity of LPG, acetone, toluene, and ethanol gas for sensor 2 wt% CdS–TiO_2_.

**Fig. 10:**
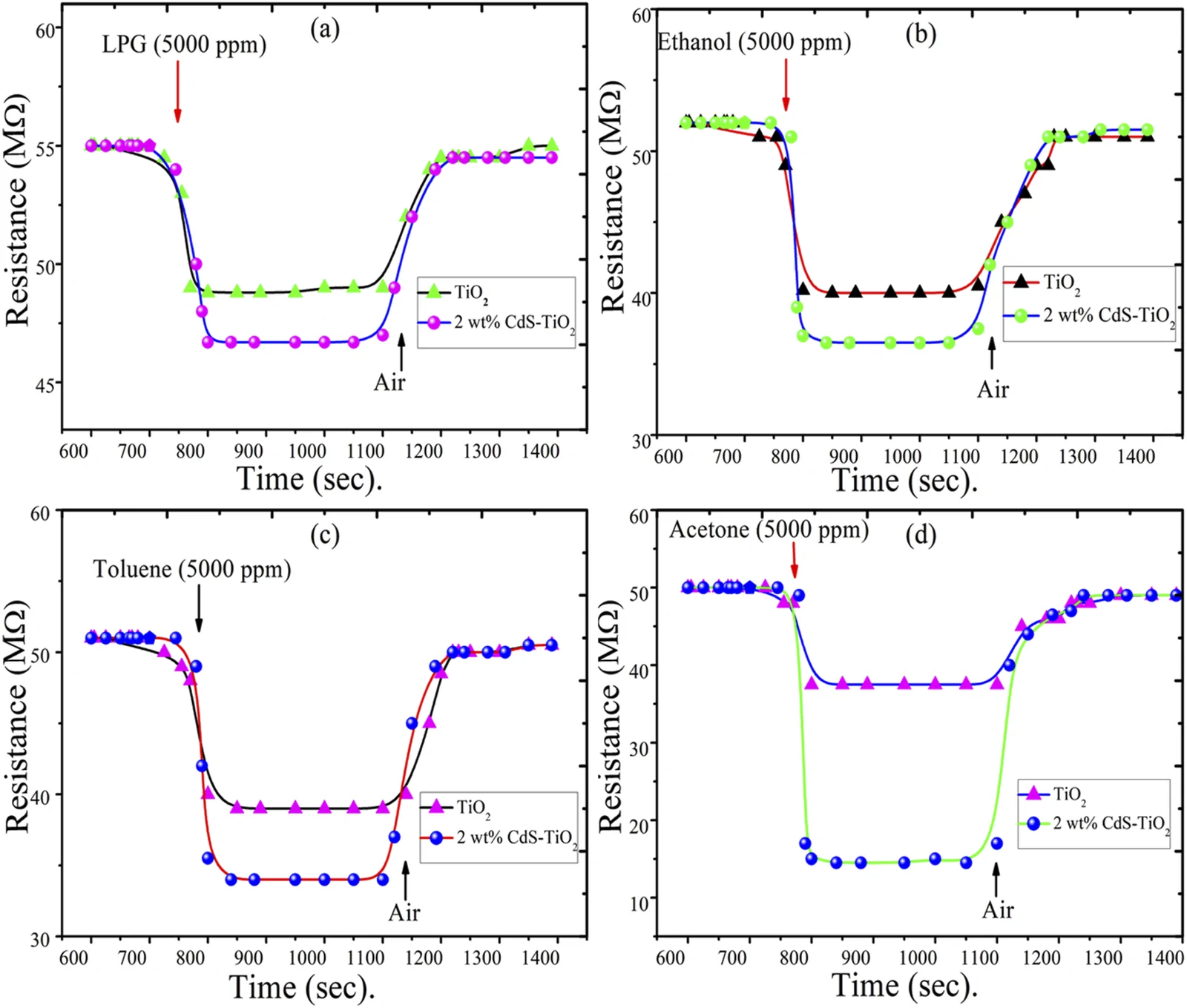
Resistance *vs.* time of sensors TiO_2_ and 2 wt% CdS–TiO_2_ at room temperature (300 K) for (a) LPG, (b) ethanol, (c) toluene, and (d) acetone gas.

Additionally, using eqn (14), a plot of log(*C*) *vs.* log[log(*R*_a_ − *R*_g_)/*R*_a_] yields a straight line, with slope *b*′ and intercept log(*B*′). The corresponding slope and intercept values are shown in Fig. 11 and summarised in Table 3. These results are in good agreement with the theoretical model and consistent with earlier studies.^57^ An elevated long-range temperature field favours the formation of fine branches during film growth. At higher substrate temperatures, the deposited films generally display thinner branches, reduced fractal density, and lower fractal dimensions. These structural features lead to a lower resistance state at the junctions, which can be more readily perturbed by an applied voltage, thereby enhancing sensor sensitivity. Sensors with fractal architectures have shown superior performance compared to conventional counterparts. This indicates that incorporating fractal geometries into the macro- and microstructural design of gas sensors could further boost sensing efficiency. To realise this potential, a detailed understanding of the mechanisms governing fractal formation, as well as the impact of deposition techniques and processing parameters on fractal morphology and dimensionality, is crucial.

**Fig. 11:**
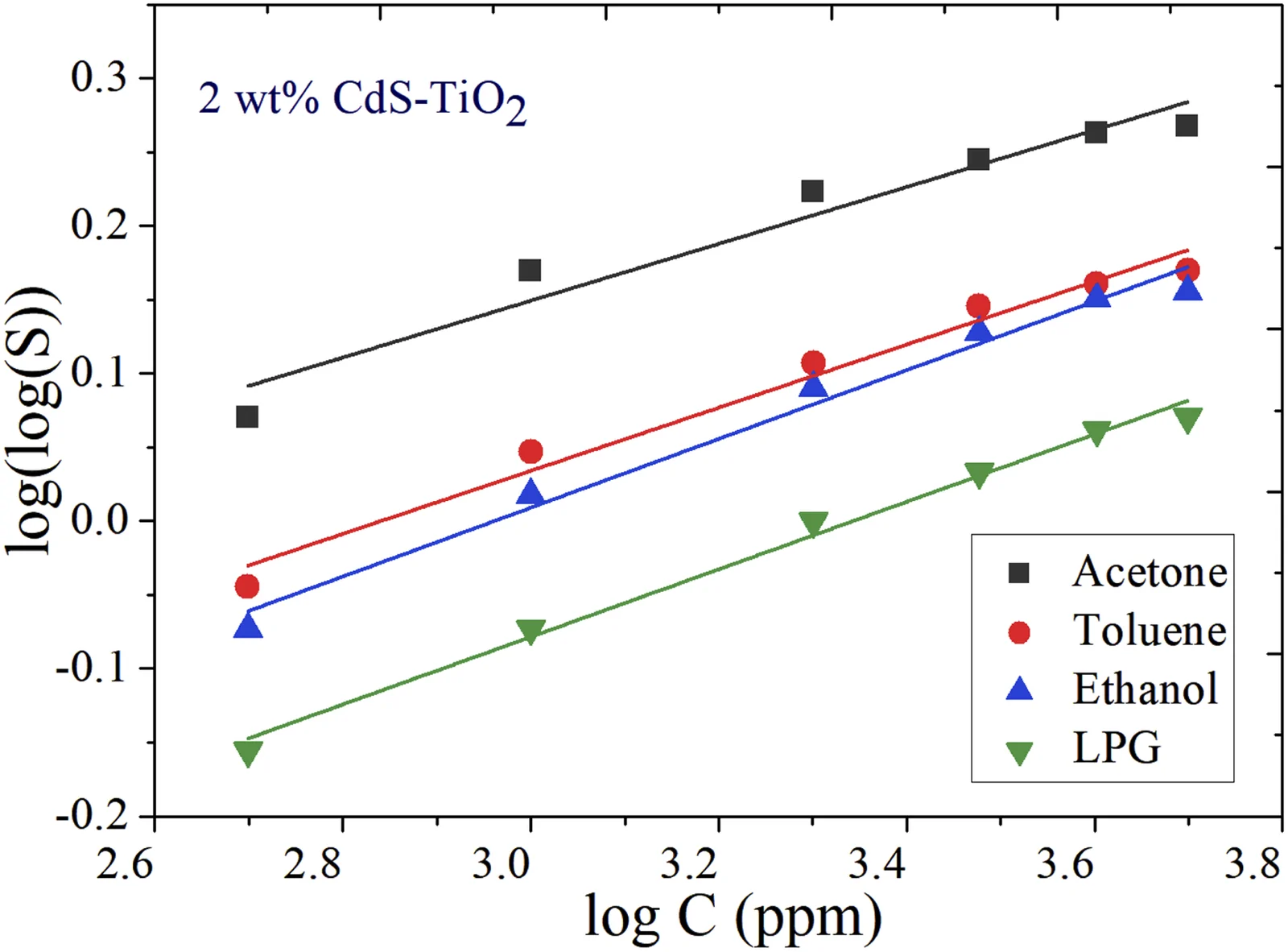
The plot of log(log(*s*)) *vs.* log *C* for test gas (0–5000 ppm) for 2 wt% CdS–TiO_2_.

**Table 3:** Linear fitting parameters and sensing limits (LOD) for ethanol, LPG, toluene, and acetone

Test gases	Slope (*s*)	S.D. (*σ*)	*R* ^2^	LOD (ppm)
Ethanol	0.23312	0.05123	0.98374	0.725
LPG	0.22885	0.03456	0.99121	0.498
Toluene	0.21327	0.05199	0.97891	0.804
Acetone	0.29244	0.01498	0.99198	0.169

The limit of detection (LOD) is defined as the lowest concentration of test gases that can be reliably detected by a sensor. It represents the point where the sensor signal can be clearly distinguished from background noise. The LOD is calculated using the following formula:^58^17
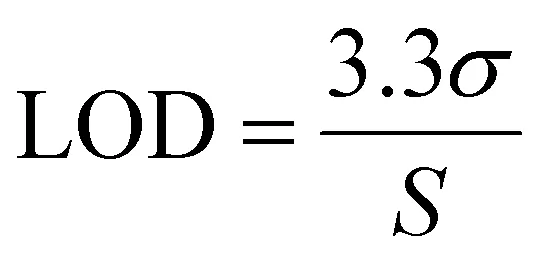
where *σ* represents the standard deviation of the intercept, and *S* represents the slope of the sensor response for a small change in concentration. The calculated LOD for the test gas is presented in Table 3. It is revealed from Table 3 that acetone exhibits the highest slope (*S* = 0.29244), indicating superior sensor response compared to LPG, ethanol, and toluene. Furthermore, the correlation coefficient (*R*^2^) for acetone and LPG is near unity, confirming an excellent linear relationship between gas concentration and sensor response. The result demonstrates that a 2 wt% CdS–TiO_2_ sensor is a highly reliable and promising candidate for selective gas sensing of acetone.

The Hurst exponent of energetic particle self-assisted deposition films in the larger scan range is in the range of 0.015–0.36. The results indicated that there are two fractal regions with different Hurst exponents in films. The fractal behaviour in the smaller range stood for grain features, while that in the larger range revealed the correlation among different grains. The variation of average Fractal dimension and Hurst exponent with grain size and the response of sensors TiO_2,_ 1 wt% CdS–TiO_2,_ and 2 wt% CdS–TiO_2_ with Hurst exponent is shown in Fig. 12. In contrast, fractal analysis captures multi-scale surface irregularity and complexity, which directly influence adsorption sites and gas surface interactions. The fractal dimension, therefore, serves as a more sensitive descriptor for correlating surface structure with sensing performance. Similar approaches have been suggested to provide deeper insight into surface-controlled processes.^59,60^

**Fig. 12:**
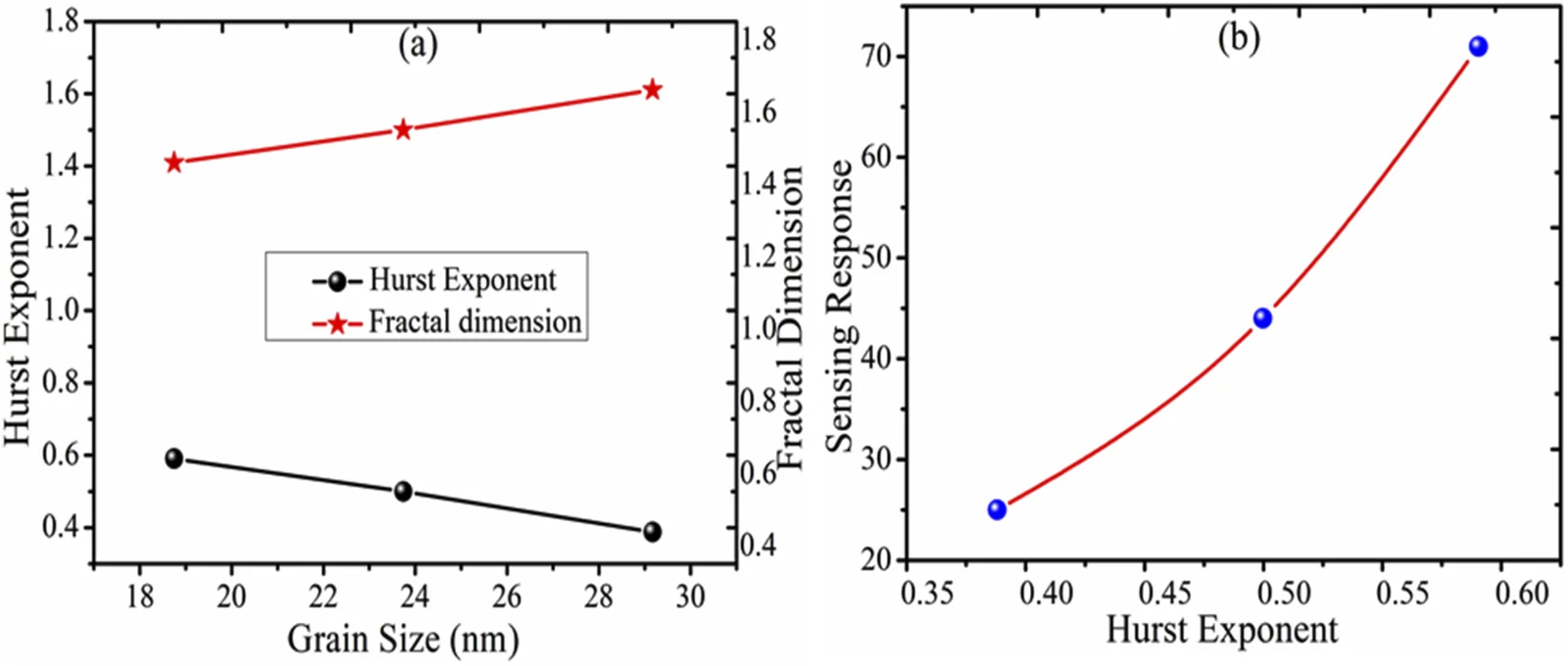
(a) Variation of average Fractal dimension and Hurst exponent with grain size (left side) and (b) response of sensors TiO_2,_ 1 wt% CdS–TiO_2,_ and 2 wt% CdS–TiO_2_ with Hurst exponent (right side).

### 5.4. Effect of relative humidity on sensing properties

Relative humidity (RH) plays a key role in determining gas sensor performance, particularly for room-temperature operation. The CdS–TiO_2_ chemiresistive sensor shows that, for most gases, increasing RH leads to a gradual decline in response, indicating that higher humidity negatively affects sensing ability. The performance of a CdS–TiO_2_ chemo-resistive sensor at various relative humidity (RH) values for various gases is shown in detail in Fig. 13. This effect becomes more pronounced at higher RH levels (70–90%). At low humidity (10% RH), the sensor exhibits maximum response to acetone (21.3%), followed by ethanol (12.2%), toluene (9.3%), and the lowest response to LPG (8.2%), demonstrating its selectivity toward acetone. The results also highlight specific RH thresholds where sensor performance significantly drops, which is important for practical applications. Since measurements were conducted under ambient conditions without humidity control, the results may include interference from moisture, as humidity is known to influence adsorption, reaction kinetics, and charge transfer processes.^61,62^

**Fig. 13:**
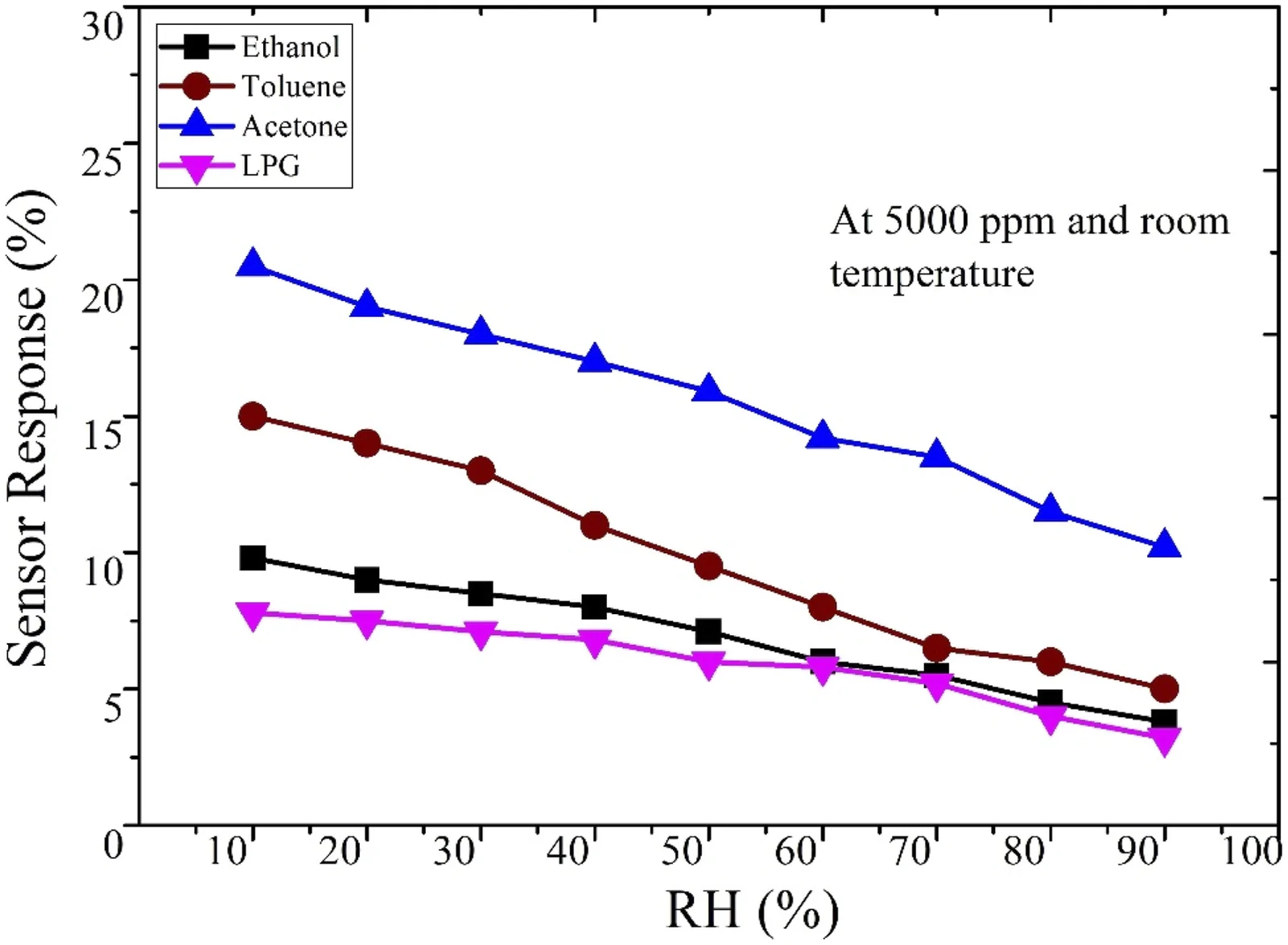
Sensor responses of the 2 wt% CdS–TiO_2_ sensors toward ethanol, toluene, acetone, and LPG at different relative humidity levels.

### 5.5. Sensing mechanism

The sensing mechanism for the CdS–TiO_2_ chemisorption sensor for sensing acetone, ethanol and Toluene can be explained by surface-adsorbed oxygen. In air, the sensor comes into contact with the environment to generate oxygen species O^−^_2,_ which are shown in Fig. 14. These oxygen species react with the test gas (acetone, ethanol, LPG and toluene) to release more electrons, resulting in a narrow depletion layer to decrease the resistance.^63^Test gases + O_2_^−^ (ads.) → CO_2_ + e^−^In the case of ethanol, toluene and acetone, the probable reaction is given byCH_3_COCH_3_ + 4O_2_^−^ (ads.) → 3CO_2_ + 3H_2_O + 4e^−^C_2_H_5_OH + 3O_2_^−^ (ads.) → 2CO_2_ + 3H_2_O + 3e^−^C_7_H_8_ + 9O_2_^−^ (ads.) → 7CO_2_ + 4H_2_O + 9e^−^

**Fig. 14:**
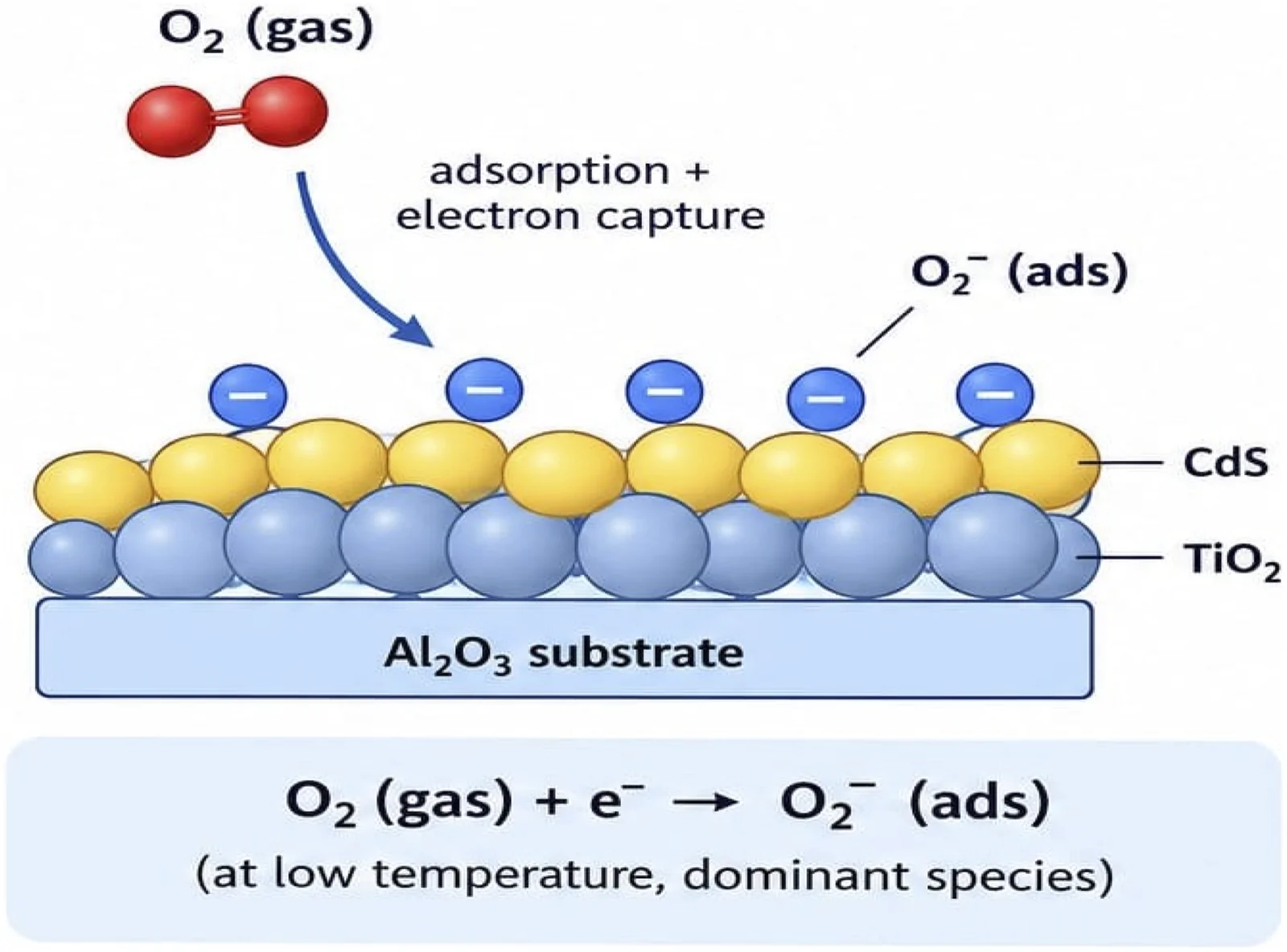
Schematic diagram of oxygen species for the CdS–TiO_2_ sensor.

Among the test gases, acetone exhibits the highest response at room temperature compared to ethanol, LPG and toluene. The enhanced response to acetone can be attributed to its strong interaction with the surface adsorption and charge transfer process at the sensing surface. When acetone contacts oxygen species, the oxygen is consumed, and electrons are released back into the semiconductor. This reduces the depletion layer and causes a decrease in resistance.

## 6. Conclusion

The nanostructure of thick films strongly affects gas-sensing performance, with fractal-like morphologies enhancing surface area. In the present study, we report on TiO_2_ and CdS-modified TiO_2_ thick film sensors. The prepared TiO_2_ films were characterised using AFM and FESEM, and their sensing properties toward VOC gas. The films with lower fractal density and dimension exhibit a reduction in resistance, improving sensor response. Hurst exponent analysis shows that 2 wt% CdS–TiO_2_ films display persistent growth behaviour (*H* > 0.5), while lower CdS content results in anti-persistent behaviour (*H* < 0.5), indicating that controlled doping influences surface evolution. The sensing behaviour of the fabricated film was tested at room temperature in air for ethanol, LPG, toluene and acetone. The results found that the highest response (71%), higher selectivity (47.7%), fast response (50 s) and highly reliable for the 2 wt% CdS–TiO_2_ sensor towards acetone gas over LPG, ethanol, and toluene. Overall, fractal geometry provides an effective approach for optimising film morphology and relates to the sensing properties of VOCs in next-generation gas sensors.

## Conflicts of interest

The authors declare that they have no conflicts of interest related to the publication of this research work.

## Data Availability

Data will be made available on request.
