# Correction: Surface morphology-driven gas sensing performance of CdS-modified TiO_2_ thick film: a study based on fractal concepts

**DOI:** 10.1039/d6ra90175a

**Published:** 2026-11-13

**Authors:** Ankit Kumar Vishwakarma, Ajaya Kumar Sharma, Ram Pratap Yadav, Pradip Kumar Priya, Madhavi Yadav, Lallan Yadava

**Affiliations:** a Department of Physics, Rajendra College, Jai Prakash University Chapra 841301 India kv.ankit92@gmail.com; b Department of Physics, Deen Dayal Upadhyaya Gorakhpur University Gorakhpur 273009 India; c Department of Physics, Allahabad Degree College, University of Allahabad Prayagraj-211003 India aurampratap@gmail.com; d Department of Physics, Ewing Christian College, University of Allahabad Prayagraj 211003 India; e Department of Physics, B. R. M. College, Munger University Munger 841301 India

## Abstract

Correction for ‘Surface morphology-driven gas sensing performance of CdS-modified TiO_2_ thick film: a study based on fractal concepts’ by Ankit Kumar Vishwakarma *et al.*, *RSC Adv.*, 2026, **16**, 64699–64714, https://www.doi.org/10.1039/d6ra06387j.

The authors regret the misspelling of author Madhavi Yadav in the author list, omitting the corresponding-author status of author Ram Pratap Yadav, and including an email address in an incorrect affiliation.

The correct author list and affiliations are as shown here.

The Royal Society of Chemistry apologises for these errors and any consequent inconvenience to authors and readers.

